# An NSP2-MYB module orchestrates flavonoid biosynthesis and nodule symbiosis

**DOI:** 10.1016/j.cub.2026.01.013

**Published:** 2026-02-04

**Authors:** Jin-Peng Gao, Chongjing Xia, Chai Hao Chiu, Qingchao Chen, Suyu Jiang, Xiaotian Wu, Wenjie Liang, Jongho Sun, Min-Yao Jhu, Jiangqi Wen, Ertao Wang, Jeremy D. Murray, Giles E.D. Oldroyd

**Affiliations:** 1 Crop Science Centre, Department of Plant Sciences, University of Cambridge, Cambridge CB3 0LE, UK; 2 Medical Research Council Laboratory of Molecular Biology, Cambridge CB2 0QH, UK; 3 Key Laboratory of Plant Carbon Capture, Chinese Academy of Sciences, JIC Centre of Excellence for Plant and Microbial Science, CAS Center for Excellence in Molecular Plant Sciences, Chinese Academy of Science, Shanghai 200032, China; 4 Department of Plant and Soil Sciences, Oklahoma State University, Stillwater, OK 74078, USA; 5 New Cornerstone Science Laboratory, Key Laboratory of Plant Carbon Capture, CAS Center for Excellence in Molecular Plant Sciences, Chinese Academy of Sciences, Shanghai 200032, China; 6 John Innes Centre, Norwich Research Park, Norwich NR4 7UH, UK

## Abstract

Flavonoids, produced by the plant under nutrient stress, are required to initiate the legume-rhizobia symbiosis through the activation of rhizobial *nod* genes. Notwithstanding the central role of flavonoids in nodulation, their transcriptional regulation remains poorly understood. Here, we show that the *nodulation signaling pathway 2* (*NSP2*) is required for transcriptional activation of flavonoid biosynthesis genes during nodulation in *Medicago truncatula*. Furthermore, MYB40, a legume-specific MYB transcription factor, is induced by rhizobia in the root epidermis. MYB40 directly binds to flavonoid biosynthetic gene promoters and is required for normal levels of nodulation. Biochemical and genetic evidence reveal that NSP2, not NSP1, interacts with MYB40 during rhizobial infection to strongly upregulate the symbiotic gene *chalcone O-methyltransferase 1* in a manner dependent on MYB40 binding sites. Moreover, the overexpression of *MYB40* and a microRNA-resistant *NSP2* variant enhances nodulation under suboptimal rhizobial availability, suggesting this module fine-tunes symbiosis efficiency. Additionally, flavonoid regulation by NSP2 and MYB40 appears to facilitate arbuscular mycorrhizal colonization under nutrient starvation. Together, our findings establish an NSP2-MYB40 module that integrates symbiotic signaling with metabolic reprogramming, representing an evolutionary innovation for optimizing nitrogen acquisition in dynamic environments.

## Graphical abstract

**Graphical abstract:**
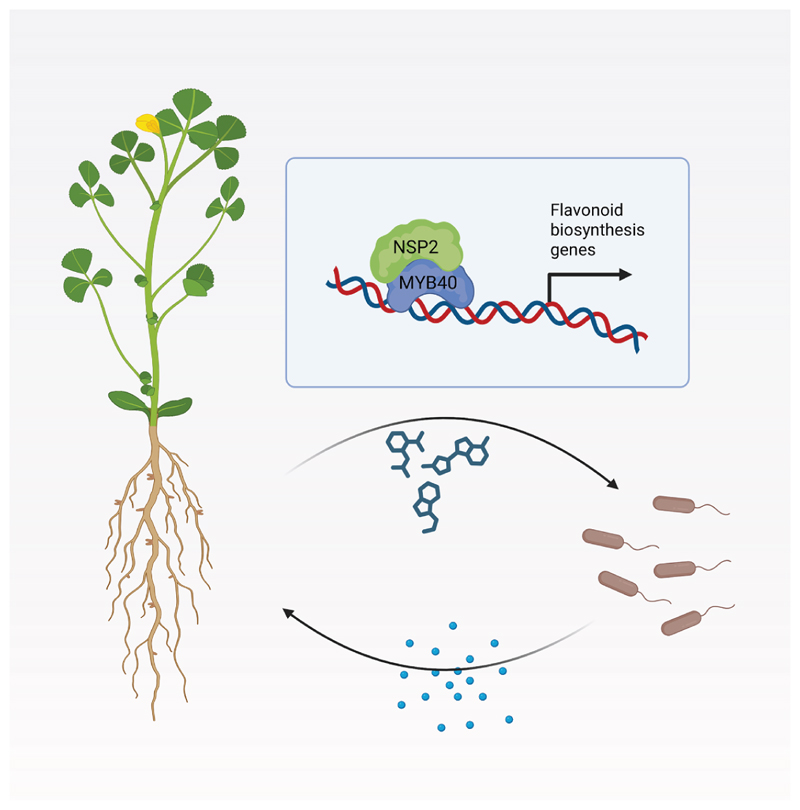

## Introduction

Plants employ adaptive strategies to survive in variable environments. Beneficial microbial symbioses enhance nutrient acquisition and sustain host fitness in fluctuating conditions.^1^ The wide-spread arbuscular mycorrhizal (AM) symbiosis enhances plant phosphorus (P) uptake through extensive hyphal networks,^2^ while legumes engage in root nodule symbiosis with rhizobia to fix nitrogen (N).^3,4^ The establishment of these endosymbioses involves the exchange of chemical signals,^5–8^ activation of the symbiosis signaling pathway,^9,10^ and intracellular microbial colonization.^11–14^ Through these mutualistic symbioses, plants obtain critical nutrients in exchange for photosynthetically fixed carbon sources.^15,16^

Flavonoids are plant metabolites derived from the phenylpropanoid pathway.^17^ Under N-limiting conditions, flavonoid biosynthesis is upregulated,^18^ and they play multifaceted roles in shaping plant-microbe interactions.^19,20^ Elevated phenylpropanoid and flavonoid levels were associated with mycorrhizal colonization and nutrient acquisition in *Medicago truncatula* and wheat.^21^ Flavonols increased AM fungal spore germination and root colonization,^22,23^ although the underlying mechanisms are unclear. Flavonoids are best known for their role in the legume-rhizobia symbiosis. During this process, flavonoid biosynthesis and accumulation are highly specific for certain cell types. Specific chalcones and flavones derived from the legume root epidermis stimulate rhizobia to produce lipochitooligosaccharides,^24–27^ known as Nod factors, which trigger symbiotic calcium spiking and downstream transcriptional responses.^10,28^ Silencing of *chalcone synthase* (*CHS*) nearly abolished nodulation,^25,29^ while *chalcone-O-methyltransferase* (*ChOMT*) was preferentially expressed in root hairs and promoted nodulation in *M. truncatula*.^26^ Furthermore, flavonols spatially regulate auxin transport to facilitate cortical nodule organogenesis.^29,30^

Flavonoid production is tightly regulated by hierarchical transcriptional networks, with MYB family transcription factors playing a central role in forming diverse protein complexes to spatiotemporally modulate flavonoid accumulation during development and under abiotic and biotic stresses.^31–33^ However, current knowledge primarily stems from pioneering studies initially in snapdragon (*Antirrhinum majus*)^34^ and later in *Arabidopsis thaliana*,^35^ non-leguminous species that lack the nitrogen-fixing symbiosis. By contrast, lineage-specific diversification in flavonoid biosynthetic genes emerged during Leguminosae evolution to facilitate the rhizobial symbiosis.^36^ In soybean (*Glycine max*), GmMYB12L and GmMYB12B2 differentially regulate flavonoid and isoflavone biosynthesis in shoots and roots, respectively, coordinating nodulation in response to ultraviolet B (UV-B) radiation.^37^ Nodule inception (NIN), the master regulator of nodulation, was recently shown to control isoflavone metabolic genes in *M. truncatula*.^38^ Nevertheless, the specialized regulatory mechanisms governing flavonoid biosynthesis during legume nodulation remain to be fully elucidated.

GRAS (GAI, RGA, and SCR) proteins are plant-specific regulators that function in diverse biological processes, including gibberellin signaling, root patterning, and microbial symbioses.^39^ Among GRAS family members, *nodulation signaling pathway 1* (*NSP1*) and *NSP2* were initially demonstrated to promote the expression of early nodulation genes, including *NIN*.^40–44^ Later studies established their roles in AM symbiosis.^45,46^ *NSP1/NSP2* regulate the biosynthesis of strigolactones,^47–49^ which are essential signaling molecules that stimulate AM fungal spore germination and hyphal branching under P-limiting conditions.^50^ However, many small molecule metabolites downstream of *NSP1/NSP2* remain uncharacterized. Strigolactones are dispensable for nodulation,^48,51^ suggesting that *NSP1/NSP2* may regulate distinct metabolites for rhizobial symbiosis.^52^ Interestingly, recent evidence from comparative transcriptomics suggests that GRAS proteins have been implicated in flavonoid production as part of adaptive responses to environmental cues.^53^ In this study, we identify a N/P-responsive symbiosis regulatory mechanism in which NSP2 interacts with MYB40, a nodulation-induced transcription factor, to directly upregulate flavonoid biosynthetic genes in *Medicago truncatula*. This NSP2-MYB40 module integrates symbiotic signaling and metabolic reprogramming, thereby optimizing nitrogen acquisition in fluctuating environments.

## Results

### Identification of flavonoid biosynthesis genes induced during nutrient starvation and rhizobial symbiosis

**Figure 1:**
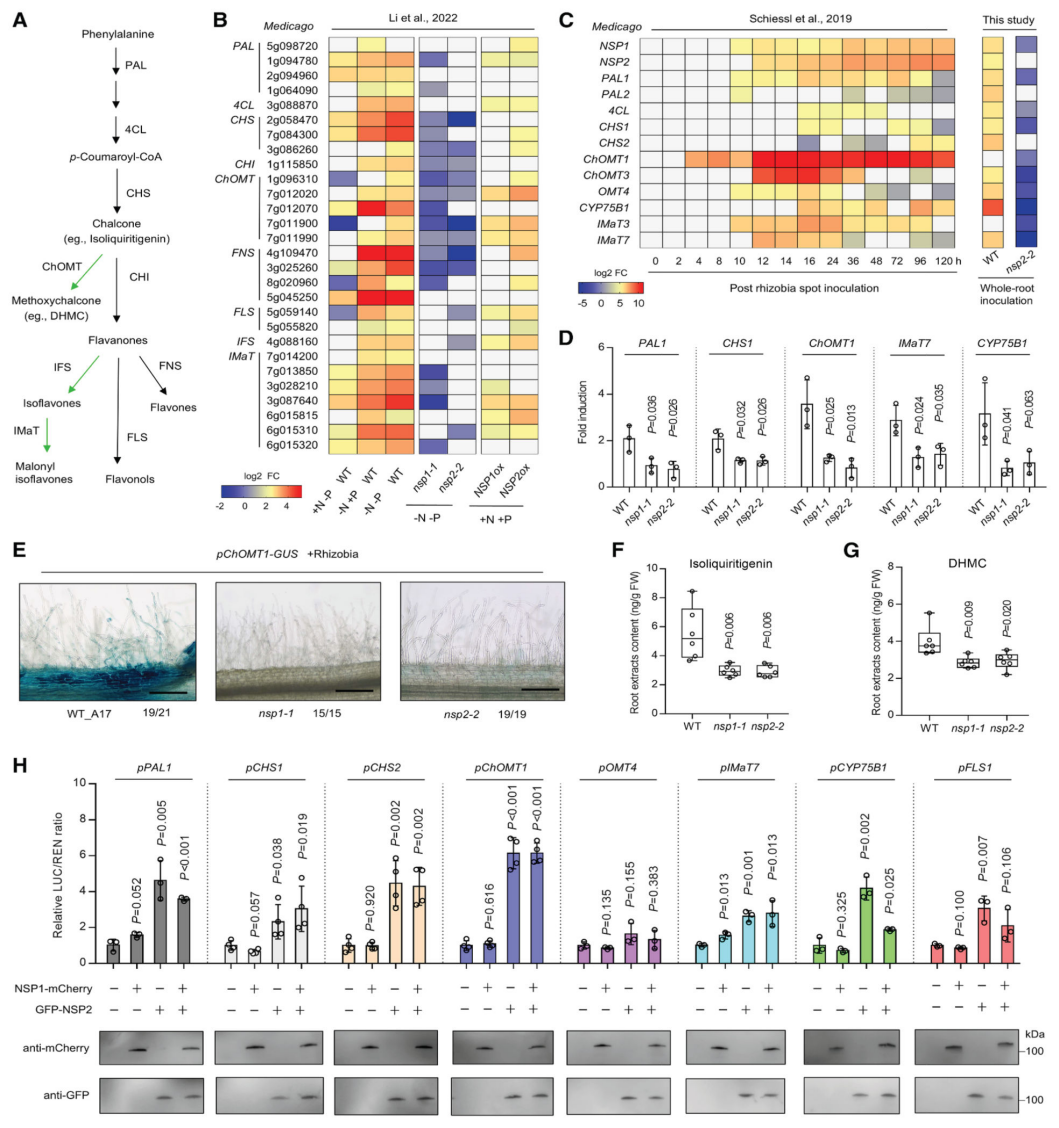
Flavonoid biosynthesis genes are induced during nutrient starvation and nodulation, with a subset exhibiting *NSP1/NSP2*-dependent regulation (A) Schematic representation of the core flavonoid biosynthetic pathway. Key enzymes shown include PAL, phenylalanine ammonia lyase; 4CL, 4-coumarate:coenzyme A (CoA) ligase; CHS, chalcone synthase; CHI, chalcone isomerase; IFS, isoflavone synthase; FNS, flavone synthase; and FLS, flavonol synthase. ChOMT is chalcone O-methyltransferase (OMT), and IMaT is isoflavone malonyl-CoA acyltransferase (MaT). ChOMT, IFS, and IMaT are primarily found in legumes and are indicated in green. (B) Heatmaps showing selected phenylpropanoid and flavonoid biosynthetic genes regulated by *NSP1*/*NSP2* in response to nitrogen (N) and phosphorus (P) starvation and activated by *NSP* overexpression in *Medicago truncatula*. Genes involved in phenylpropanoid and flavonoid biosynthetic pathways are annotated. +N−P, −N+P, and −N−P represent the expression of these genes in wild-type plants by comparing −N or/and −P conditions to +N+P. The *nsp* mutants show gene expression in *nsp* mutants compared with wild-type plants under nutrient depletion, while *NSPox* shows *NSP* overexpression in roots compared with wild type under nutrient-replete conditions. The concentrations used were defined as follows: −N−P, no NO^3−^ and no PO_4_^3−^; −N+P, no NO^3−^ and 0.5 mM PO_4_^3−^; +N−P, 5 mM NO^3−^ and no PO_4_^3−^; and +N+P, 5 mM NO^3−^ and 0.5 mM PO_4_^3−^. Color scale represents log2 fold change. Data from Li et al.^48^ (C) Heatmap showing selected genes induced after rhizobia inoculation. Data in the left panel from Schiessl et al.^54^; *M. truncatula* ecotype jemalong and Sm2011 were used. The right panel shows transcriptome profiling of *nsp2-2* mutant compared with the wild-type control (A17) under whole-root inoculation conditions at 7 days post inoculation (dpi) with Sm2011 on Fahraeus plant agar plates. See also Data S1 and S2. (D) Expression analysis of a subset of flavonoid biosynthetic genes in wild type (A17), *nsp1-1*, and *nsp2-2* at 3 dpi in soil. Data are presented as mean ± SD (*n* = 3). Statistical significance was determined by Student’s *t* test. (E) Promoter activity of *ChOMT1* visualized by GUS (blue) in wild-type A17, *myb40-1, nsp1-1*, and *nsp2-2* mutants after rhizobia inoculation. The numbers below the images indicate the numbers of roots having a pattern similar to the one shown in the figure as representative among the total number of analyzed roots. Scale bars, 200 μm. (F and G) Analysis of isoliquiritigenin and 4,4′-dihydroxy-2′-methoxychalcone (DHMC) in roots of wild type (A17), *nsp1-1*, and *nsp2-2* at 3 dpi. Boxes show the first quartile, median, and third quartile; whiskers show minimum and maximum values; and dots show data points (*n* = 6). FW, fresh weight. Statistical significance was determined by Student’s *t* test. (H) Transactivation assays in *Nicotiana benthamiana*. The luciferase (LUC) activity induced upon the co-expression of *NSP1* and/or *NSP2* with different promoter-LUC reporters was analyzed, and the LUC activity was normalized to the Renilla (REN) activity. Data are presented as mean ± SD (*n* = 3–4 biological replicates). Statistical significance was determined by Student’s *t* test, with *p* values indicated above the columns relative to empty vector (EV) control. The western blot below shows that both proteins are expressed. Experiments were repeated three times with similar results. See also Figures S1 and S6.

To investigate the regulation of flavonoid biosynthesis genes, we first analyzed a preexisting comparative RNA sequencing (RNA-seq) dataset profiling *M. truncatula* grown under different nutrient conditions (+N+P, +N −P, − N+P, and −N−P). ^48^ We identified a set of genes encompassing many, if not all, of the key components within the phenylpropanoid and flavonoid biosynthetic pathways that are induced during nutrient starvation (Figures 1A and 1B). This collection includes both the primary rate-limiting enzymes, *phenylalanine ammonia lyase* (*PAL*) and *CHS*, and diversification enzymes such as *flavone synthase* (*FNS*) and *flavonol synthase* (*FLS*) that govern flavonoid structural variation. Under P-deficiency (+N−P), the biosynthetic genes, including *ChOMT* and *isoflavone malonyltransferase* (*IMaT*), were induced yet also exhibited suppression in some instances compared with the +N-+P control. A stronger induction of most genes was observed under both N-deficiency (−N+P) and dual deficiency (−N−P) conditions (Figure 1B).

Rhizobial spot inoculation of *Sinorhizobium meliloti* 2011 (Sm2011) on the root susceptibility zone in *M. truncatula*^54^ triggered upregulation of key flavonoid metabolic genes during nodulation (Figures 1C and 1D). The expression of *PAL1/2* was induced, whose *Lotus japonicus* ortholog, *LjPAL1*, plays dual roles in plant defense and rhizobial symbiosis.^55^ The induction of *CHS1/2* is consistent with their established role in root endosymbiosis.^25^ *ChOMT1/3* show the strongest induction and have been demonstrated to promote nodulation in *M. truncatula*.^26^ *IMaT3/7* were also induced by rhizobia, and their soybean (*Glycine max*) homolog *GmMaT2* is involved in nodulation by modifying the synthesis of isoflavones.^56^ Furthermore, *CYP75B1/2* encode a cytochrome P450, potentially catalyzing luteolin production. As an established *nod* gene inducer,^57,58^ application of luteolin can enhance nodulation in *M. truncatula*.^25^

Analysis of available single-cell RNA-seq data^59,60^ revealed cell-type-specific induction patterns of flavonoid biosynthetic genes during symbiotic interactions (Figures S1A and S1B). *PAL1* and *IMaT3/7* exhibit higher expression levels in cortical cells, while *CYP75B1* was more abundant in the epidermis. Notably, *ChOMT1* showed the strongest and most preferential induction in epidermal cells by rhizobia or Nod factor, which was further validated through histochemical β-glucuronidase (GUS) staining with a *ChOMT1* promoter-GUS reporter construct (Figure 1E).

These results suggest that many flavonoid biosynthesis genes are induced under both nutrient starvation and symbiosis, highlighting their dual role as metabolic mediators of nutrient stress adaptation and symbiotic signaling.

### *NSP1* and *NSP2* promote the expression of flavonoid biosynthesis genes

*NSP1* and *NSP2* are master integrators of nutrient signaling (N/P starvation) and symbiosis activation^47,48,61^; therefore, we focused on their potential roles in flavonoid regulation. We observed that the induction of many flavonoid biosynthetic genes was partially or completely suppressed in *M. truncatula nsp1* and *nsp2* mutants compared with the wild type under nutrient-deficient (−N−P) conditions (Figure 1B). Conversely, overexpression of *NSP1* or *NSP2* activated the expression of some flavonoid genes, even under nutrient-replete (+N+P) conditions (Figure 1B). Furthermore, rhizobia-induced expression of *CHS1, ChOMT1, CYP75B1*, and *IMat7* was abolished in both *nsp1* and *nsp2* mutants (Figures 1D, 1E, S1C, and S1D). Notably, the levels of isoliquiritigenin (a CHS product) and 4,4′-dihydroxy-2′-methoxychalcone (DHMC, produced by ChOMT) were significantly decreased in the roots of *nsp1* and *nsp2* mutants (Figures 1F and 1G). These results suggest that *NSP1/NSP2* transcriptionally control flavonoid metabolism during nutrient-starvation conditions and during the rhizobial symbiosis.

To determine whether *NSP1*/*NSP2* regulate these flavonoid biosynthesis genes, we performed transactivation analysis in a *Nicotiana benthamiana* heterologous system. The results revealed that *NSP2*, but not *NSP1*, was capable of transactivating the tested promoters of flavonoid pathway genes, including *PAL1, CHS1/2, ChOMT1, CYP75B1*, and *IMaT7* (Figure 1H). However, co-expression of *NSP1* and *NSP2* did not further enhance transactivation compared with *NSP2* alone (Figure 1H).

NSP1 and NSP2 form a heterodimer that directly binds to the promoters of nodulation genes and strigolactone biosynthesis genes.^43,49^ We therefore assessed whether NSP1/NSP2 are able to bind the promoters of flavonoid biosynthesis genes. We performed electrophoretic mobility shift assays (EMSA) but found no evidence for direct binding to the tested promoter regions of *PAL1, CHS1, ChOMT1, IMaT7*, and *CYP75B1* (Figures S1E and S1F). Although we cannot exclude the possibility that the NSP1-NSP2 complex can bind to flavonoid metabolic gene promoters in planta, our results suggest that NSP2 acts as a transcriptional activator of flavonoid biosynthesis genes through recruitment of other transcription factors.

### MYB40 directly regulates flavonoid biosynthesis genes to mediate nodule symbiosis

**Figure 2:**
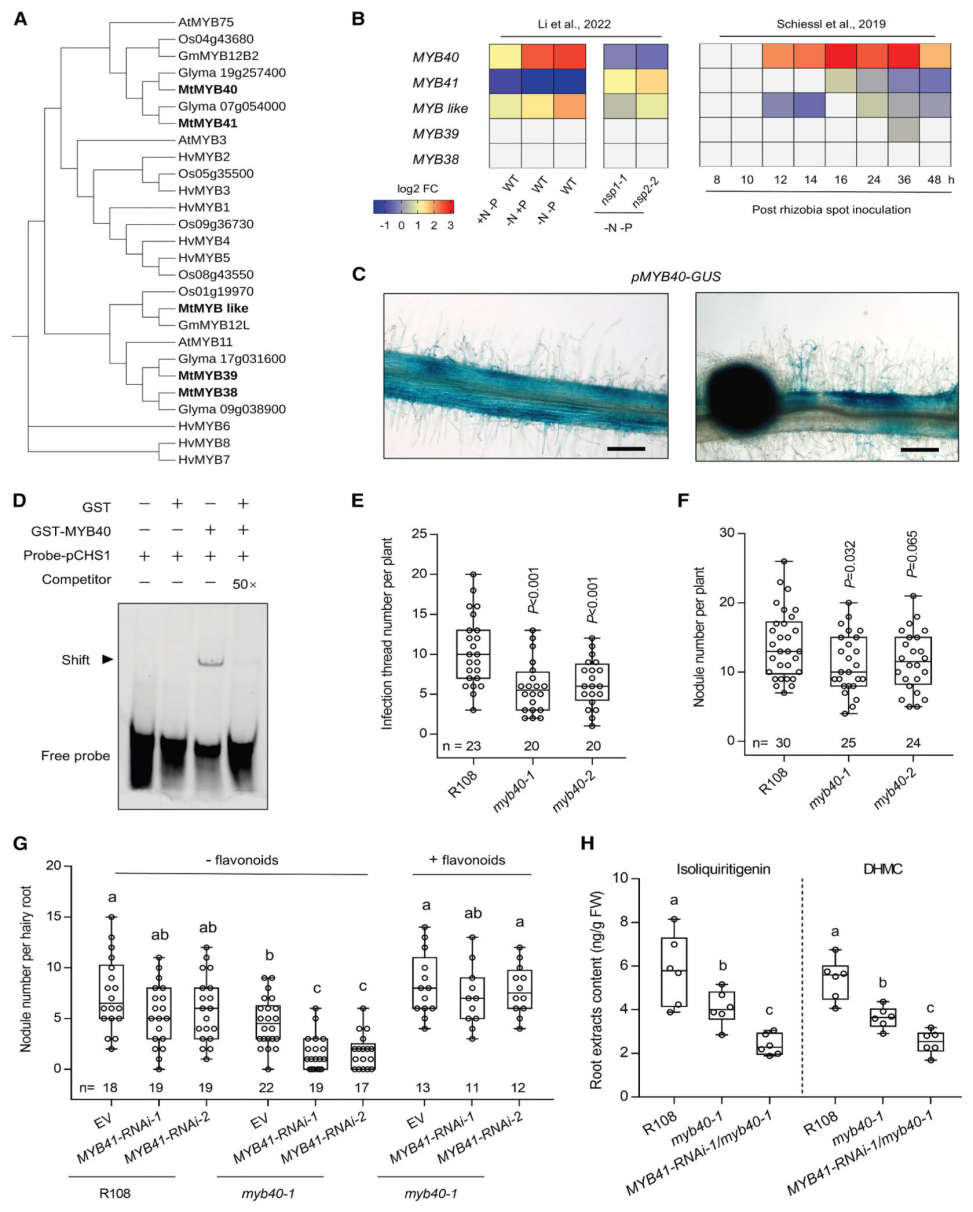
MYB40 is required for rhizobial symbiosis (A) Phylogenetic distribution of MYB40 homologs from different plant species. The phylogenetic tree was constructed using PhyML and presented using iTOL (https://ngphylogeny.fr/). See also Figure S2B. (B) Heatmaps showing the expression of the *M. truncatula MYB* genes in response to nitrogen (N) and phosphorus (P) starvation and post-rhizobia inoculation. Color scale represents log2 fold change. Data in the left and middle panels from Li et al.^48^; the concentrations used were defined as follows: −N−P, no NO^3−^ and no PO_4_^3−^; −N+P, no NO^3−^ and 0.5 mM PO_4_^3−^; +N−P, 5 mM NO^3−^ and no PO_4_^3−^; and +N+P, 5 mM NO^3−^ and 0.5 mM PO_4_^3−^. Data in the right panel from Schiessl et al.^54^; *M. truncatula* ecotype jemalong and Sm2011 were used. (C) Images showing *pMYB40:GUS* activity in epidermal cells and root nodules post-rhizobia inoculation. Scale bars, 200 μm. (D) Recombinant glutathione S-transferase (GST)-MYB40 protein binds to the promoter of *CHS1 in vitro*. The Cy5-labeled probes were incubated with GST-tagged MYB40. Competition of the binding with 50-fold unlabeled wild-type probes is shown in the last lane. A band shift indicates positive probe binding. Experiments were repeated twice with similar results. (E and F) Quantification of infection threads (E) at 5 dpi and nodules (F) at 14 dpi. Boxes show the first quartile, median, and third quartile; whiskers show minimum and maximum values; and dots show data points. Statistical significance was determined by Student’s *t* test, with *p* values indicated above the columns. (G) Quantification of total nodules in transgenic roots at 14 dpi in a 1:1 mixture of Terra Green and sand. The three bar graphs on the right show nodulation after flavonoid treatment. Letters denote statistically significant groupings analyzed by one-way ANOVA with Tukey’s test. (H) Analysis of isoliquiritigenin and DHMC in roots of R108, *myb40-1*, and *MYB41-RNAi/myb40-1* at 3 dpi. Letters denote statistically significant groupings analyzed by one-way ANOVA with Tukey’s test. See also Figures S2 and S3.

Promoter analysis of symbiosis-related flavonoid biosynthesis genes identified a conserved cis-regulatory element (Figure S2A), with predicted binding affinity for a MYB subfamily (Figures 2A and S2B). MYB transcription factors are known contributors of flavonoid biosynthesis gene regulation in other species.^62,63^ In *M. truncatula, MYB40* (Medtr7g117730), a homolog of flavonoid regulator *GmMYB12B2* (Figures 2A and S2B), shows dual induction in response to both nutrient starvation and nodulation (Figure 2B). Notably, *MYB40* is highly expressed in root hairs and the underlying cortical cells upon rhizobial inoculation (Figure 2C), exhibiting a similar expression pattern to the nodulation-induced flavonoid biosynthesis genes (Figures S1A and S1B), suggesting its potential role in their regulation. Structural prediction by AlphaFold3 suggested that MYB40 binds the promoter of flavonoid biosynthesis genes, such as *CHS1* and *ChOMT1* (Figure S2C), with moderate to high confidence (pTM = 0.79, ipTM = 0.61). This prediction was further validated by EMSA, which demonstrated direct binding of MYB40 (Figure 2D).

To investigate the role of *MYB40* in nodulation, we obtained *myb40 Tnt1* insertion mutants (NF21386 and NF9316; Figure S3A). Under normal inoculation conditions (Sm2011, OD600 = 0.1), two independent homozygous *myb40* mutants showed a significantly reduced number of infection threads compared with wild-type R108 plants (Figure 2E), while the number of nodules was only slightly reduced (Figure 2F). The induction of *CHS1* and *ChOMT1* during nodulation was impaired in the *myb40* mutants (Figure S3B). The levels of isoliquiritigenin and DHMC were also decreased in *myb40-1* roots (Figure 2G). While the impaired flavonoid levels likely reduced early symbiotic interactions with rhizobia, the residual amount appeared sufficient to support nodulation. Under lower-titer rhizobia inoculation (Sm2011, OD600 = 0.01), both *myb40* alleles exhibited statistically significant reductions in nodule number compared with wild-type controls at 14 days post inoculation (dpi) (Figure S3C). This reduced nodulation phenotype of the *myb40* mutants can be restored by external supplementation with flavonoids (Figure S3C) and complementation with the coding sequence of *MYB40* in transgenic roots (Figure S3D).

The weak phenotype of *MYB40* might result from genetic redundancy. To test this, we employed RNA interference (RNAi) to knockdown its close homolog *MYB41* (Figure S3E). Two independent RNAi constructs targeting *MYB41* in wild-type R108 caused a slight reduction in nodule number, whereas introducing *MYB41-RNAi* into the *myb40-1* mutant produced a more enhanced phenotype than either the *myb40* single mutant or the RNAi lines in the R108 (Figure 2G). This reduced nodulation could also be rescued by flavonoid supplementation (Figure 2G). Furthermore, measurement of isoliquiritigenin and DHMC revealed their levels to be most strongly reduced in the *MYB41-RNAi-1/myb40-1* transgenic roots relative to the wild-type and *myb40* single mutant (Figure 2H). These results suggest that MYB40 positively regulates flavonoid biosynthesis to modulate symbiotic nodulation in *M. truncatula*.

### NSP2 interacts with MYB40

**Figure 3:**
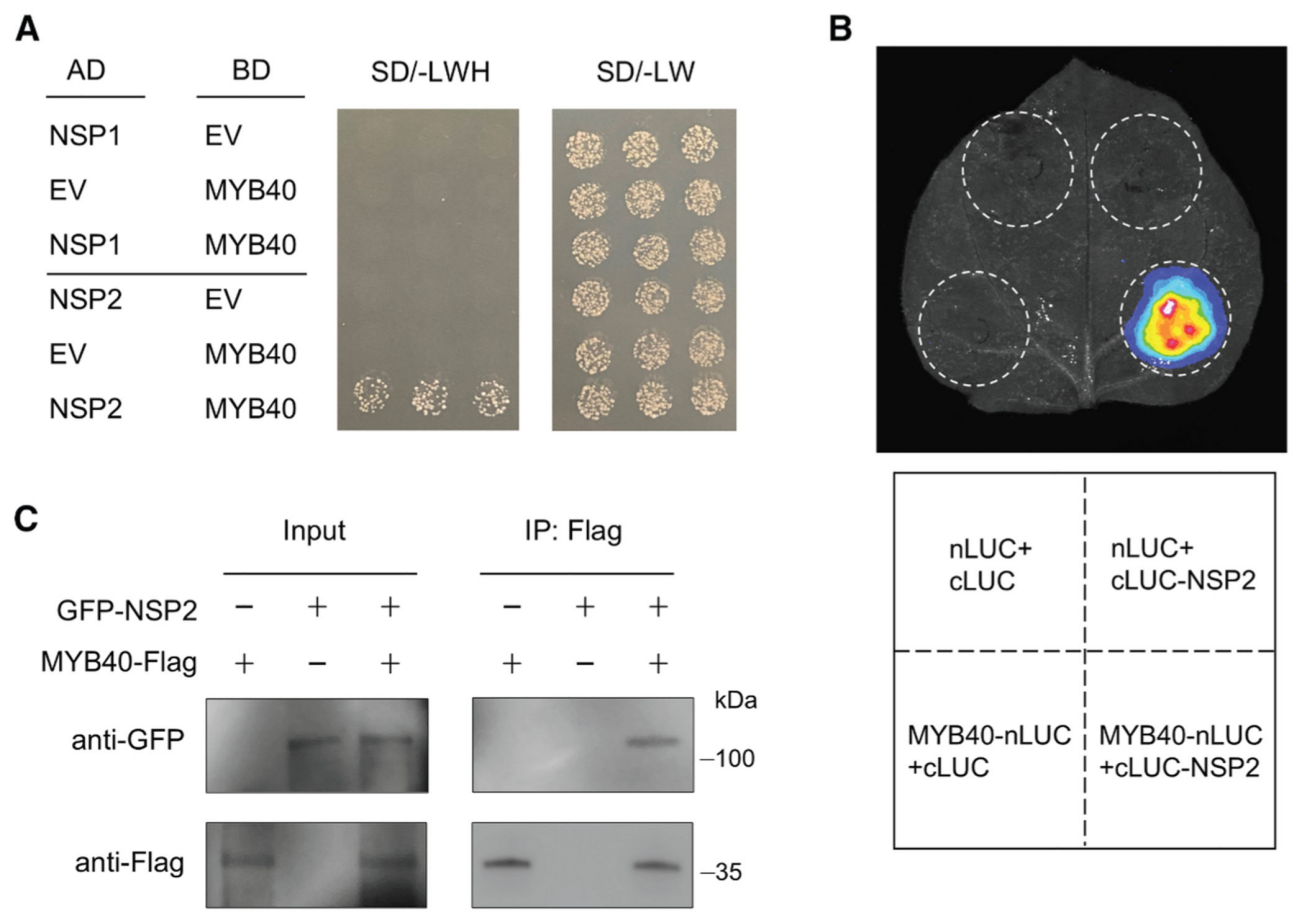
NSP2 interacts with MYB40 in yeast and in planta (A) Y2H assays between NSP1/NSP2 and MYB40. The combinations of proteins expressed in either the prey vector (pGADT7, AD) or the bait vector (pGBKT7, BD) are indicated alongside the yeast colonies. Yeast cells were plated onto SD-3/-Leu-Trp-His medium and SD-2/-Leu-Trp medium. (B) Split luciferase (LUC) complementation assays between NSP2 and MYB40. The N-terminal fragment of LUC (nLUC)-tagged MYB40 was co-infiltrated into *N. benthamiana* leaves along with the C-terminal fragment of LUC (cLUC)-tagged NSP2. (C) Coimmunoprecipitation (CoIP) assays of GFP-NSP2 and MYB40-FLAG in *M. truncatula* transgenic roots after rhizobia inoculation. Proteins were immunoprecipitated (IP) with anti-FLAG-M2 beads and analyzed by western blot using horseradish peroxidase-conjugated anti-FLAG or anti-GFP antibody. Experiments were carried out two (C) or three times (A and B) with similar results. See also Figure S4.

We hypothesized that NSP2 may act through MYB40 to activate the expression of flavonoid biosynthesis genes. To investigate potential physical associations between NSP1/NSP2 and MYB40, we performed a yeast two-hybrid assay. This revealed that NSP2, but not NSP1, can interact with MYB40 in yeast (Figure 3A). Subcellular localization analysis in *N. benthamiana* leaf cells showed co-localization of GFP-NSP2 and MYB40-mCherry in the nucleus (Figure S4A). The interaction of NSP2 and MYB40 was confirmed *in vivo* using a split luciferase (LUC) complementation assay in *N. benthamiana* (Figure 3B) and using a co-immunoprecipitation assay in *M. truncatula* transgenic roots inoculated with *S. meliloti* 2011 (Figure 3C).

NSP2 contains two leucine heptad repeat domains (LHRI and LHRII) and three conserved GRAS family domains (VHIID, PFYRE, and SAW). Structure prediction by AlphaFold3 reveals that the VHIID, PFYRE, and SAW domains of NSP2 assemble into a defined groove that accommodates the C-terminal α-helix of MYB40, with the LHR1 domain of NSP2 contacting the MYB40 N-terminal DNA-binding domain (Figure S4B), suggesting a potential dual anchoring mechanism that stabilizes the overall complex. Together, these results indicate that NSP2 physically associates with MYB40.

### NSP2-MYB40 interaction enhances the transcriptional activation of *ChOMT1* to promote nodulation

**Figure 4:**
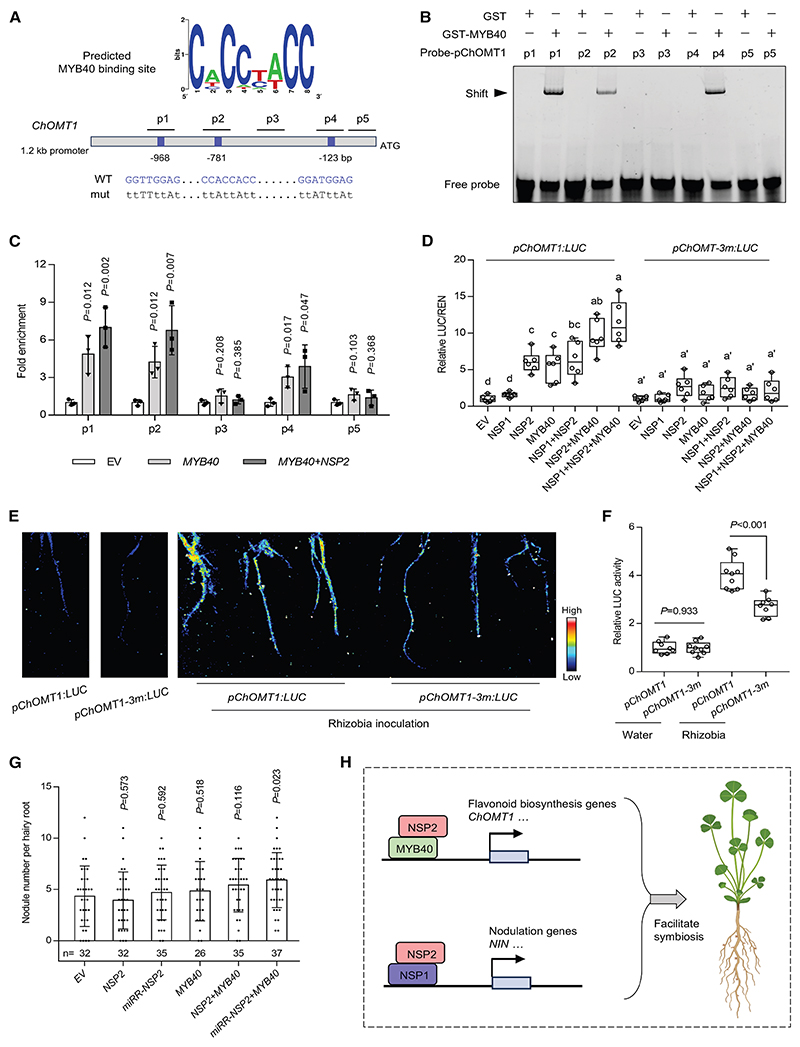
NSP2 interacts with MYB40 to enhance its transcriptional activity (A) Promoter analysis of flavonoid biosynthesis genes induced by nutrient starvation identified putative MYB40 binding sites using MEME (multiple EM for motif elicitation). Blue color box indicated the predicted MYB40 binding site in the *ChOMT1* promoter. (B) Recombinant GST-MYB40 protein binds the *ChOMT1* promoter *in vitro*. Five distinct promoter fragments (p1 to p5), as indicated in (A), were tested. Band shifts indicate binding of MYB40 to the promoter probes. (C) ChIP-qPCR analysis of MYB40 binding to the *ChOMT1* promoter in *M. truncatula* transgenic roots. ChIP assays were performed using transgenic roots expressing either *MYB40-FLAG* alone or both *MYB40-FLAG* and *GFP-NSP2*. IP was carried out using a monoclonal anti-FLAG antibody. qPCR was performed using primers to target the *ChOMT1* promoter region (p1 to p5). Statistical significance was determined by Student’s *t* test. (D) Transient dual-luciferase reporter assay in *N. benthamiana* leaves. The luciferase (LUC) activity driven by the *pChOMT1:LUC* reporter or the mutated version *pChOMT1-3m:LUC* was analyzed upon co-expression of *MYB40, NSP1*, and *NSP2*. LUC activity was normalized to the Renilla (REN) activity. Boxes show the first quartile, median, and third quartile; whiskers show minimum and maximum values; and dots show data points. Letters denote statistically significant groupings analyzed by one-way ANOVA with Tukey’s test. Experiments were repeated three times with similar results. (E) Luminescence images of *pChOMT1:LUC* reporter in *M. truncatula* transgenic roots at 2 dpi (Sm2011, OD600 = 0.01). Experiments were repeated twice with similar results. (F) Quantitative analysis of luminescence signals in transgenic roots using ImageJ. Statistically significant differences were detected by Student’s *t* test, and *p* values indicated above the box plots. (G) Nodule numbers per hairy root plant transformed with *NSP2, miRR-NSP2* (miR171h-resistant version of *NSP2*), and *MYB40* at 14 dpi (Sm2011, OD600 = 0.01). Numbers below columns represent the number of biologically independent sample sizes. Data are mean ± SD. Statistically significant differences were detected by Student’s *t* test, and *p* values indicated above the columns relative to empty vector (EV) control. Experiments were carried out twice with similar results. (H) Proposed model of NSP2-mediated regulation of flavonoid biosynthesis genes during nodulation in *M. truncatula*. NSP2 interacts with MYB40 to directly activate flavonoid biosynthesis genes, particularly *ChOMT1*. The NSP2-NSP1 heterodimer activates nodulation genes (e.g., *NIN*) and may indirectly regulate flavonoid biosynthesis genes. See also Figures S2 and S4.

In *M. truncatula*, a *chomt1/chomt3/omt2* triple mutant shows defects in nodulation, and overexpression of *ChOMT1* can promote nodulation.^26^ Notably, *ChOMT1* was highly expressed in the epidermis and showed the strongest rhizobial-induced upregulation of all the flavonoid biosynthesis genes (Figure 1C), prompting us to focus further analysis on the *ChOMT1* promoter. This promoter showed at least three predicted MYB40 binding motifs (Figure 4A). EMSA demonstrated specific binding of MYB40 to these regions, while no detectable interaction was observed with adjacent control sequences in the promoter (Figure 4B). Furthermore, the MYB40-DNA interaction could not be outcompeted with the addition of an unlabeled probe with mutations in the MYB40 binding site, while excess unlabeled wild-type probe effectively competed with the labeled promoter probes and eliminated the observed shift, indicating the specificity of the interaction (Figure S4C). To determine whether NSP2 potentiates the DNA-binding activity of MYB40, we performed EMSA using various combinations of these transcription factors with the *ChOMT1* promoter elements. However, no larger DNA-protein complex or enhanced band was observed compared with MYB40 alone (Figure S4C), potentially due to technical limitations inherent to *in vitro* conditions. We next performed chromatin immunoprecipitation (ChIP) in *M. truncatula* transgenic roots. ChIP-qPCR analysis revealed enrichment of the *ChOMT1* promoter fragments by MYB40, with further enhanced enrichment upon co-expression of *NSP2* and *MYB40* (Figure 4D). These results suggest that NSP2 may facilitate the association of MYB40 with DNA *in vivo*.

Moreover, transcriptional activation assays revealed that *MYB40* and *NSP2* together drive significantly stronger ransactivation of the *ChOMT1* promoter than *MYB40* or *NSP2* alone (Figure 4D). This suggests that the physical interaction between MYB40 and NSP2 may facilitate the activation of *ChOMT1* during nodulation. When we mutated the three identified potential MYB40 binding sites, the promoter activity was dramatically reduced in the presence of any of the tested transcription factor combinations (Figure 4D), indicating that NSP2 activation of the promoter appears to be dependent on the MYB-binding sites.

To further confirm this activation in *M. truncatula*, we generated transgenic roots expressing the *pChOMT1:LUC* reporter. These transgenic roots exhibited basal levels of luminescence under non-inoculated conditions (Figure 4E). However, upon inoculation with rhizobia (Sm2011, OD600 = 0.01), luminescence driven by the *ChOMT1* promoter significantly increased (Figures 4E and 4F). By contrast, when the three potential MYB40 binding sites were mutated in roots of composite plants, the luminescence signal was markedly reduced (Figures 4E and 4F).

Next, we examined whether overexpression of *NSP2* and *MYB40* could enhance nodulation. Since *NSP2* is post-transcriptionally regulated by microRNA miR171h,^45,64^ we included a miR171h-resistant version of *NSP2* (*miRR-NSP2*)^48^ in our analysis. Although expression of *NSP2, MYB40* alone, or their combination showed no significant difference in nodule number, the transgenic roots co-expressing *miRR-NSP2* and *MYB40* exhibited more nodules at a suboptimal rhizobial concentration (Sm2011, OD600 = 0.01) (Figure 4G).

Together, these results suggest that NSP2 activates the *ChOMT1* promoter through its association with MYB40, facilitating nodulation under suboptimal symbiotic conditions.

### The NSP2-MYB module is involved in the AM symbiosis

**Figure 5:**
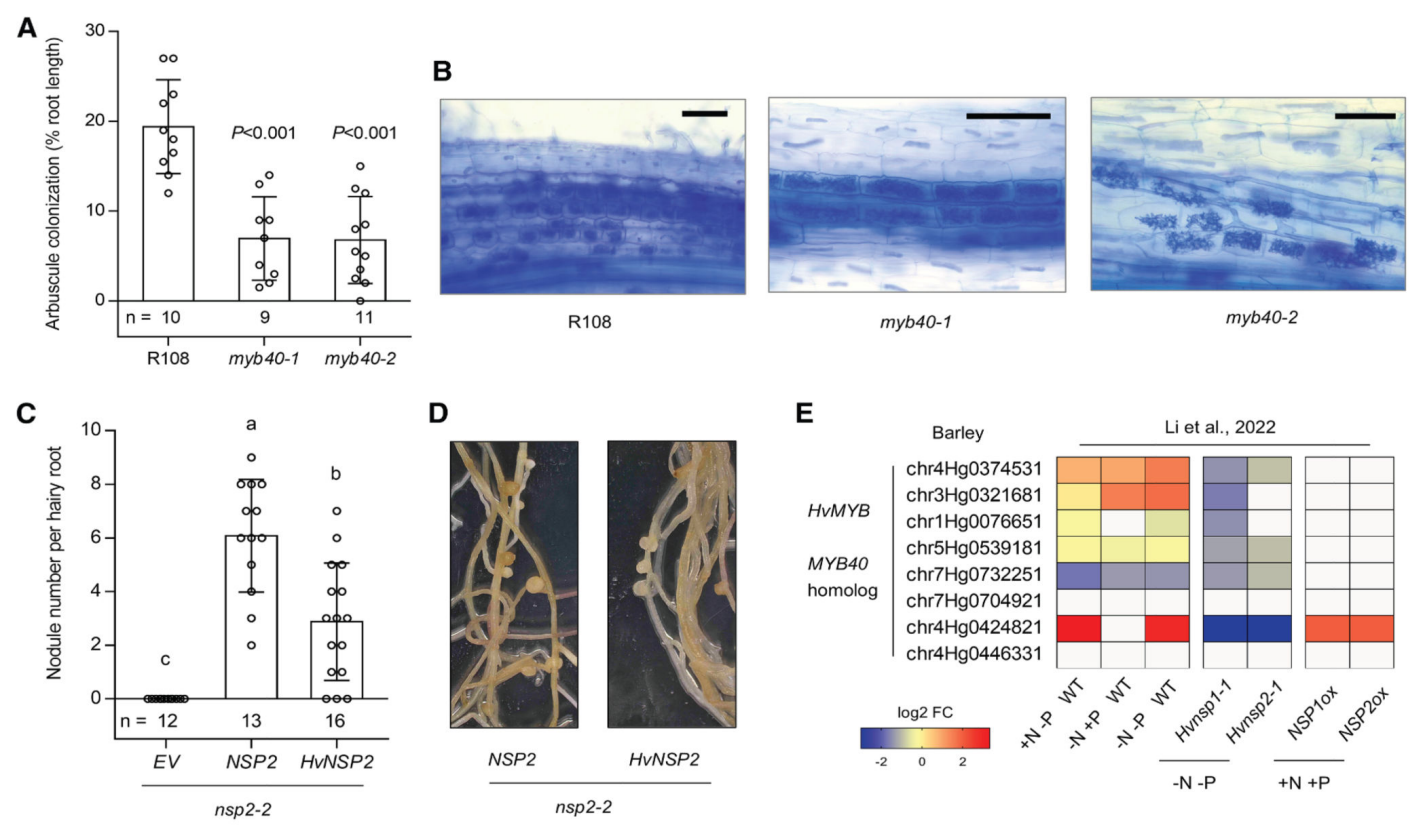
MYB40 is involved in the AM symbiosis (A) Root length colonization by AM fungi. Wild-type R108 ecotype and *myb40* mutants were co-cultivated with *Rhizophagus irregularis* assessed at 35 dpi. Plants were inoculated with 300 *R. irregularis* spores. Data are mean ± SD. Statistically significant differences were detected by Student’s *t* test, and *p* values indicated above the columns relative to R108. Numbers below columns represent the number of biologically independent sample sizes. Experiments were repeated twice with similar results. (B) Representative images of arbuscule morphology in R108 and *myb40* mutant roots. Roots were stained with trypan blue to visualize plant and fungal structures. Scale bars, 100 μm. (C) Genetic complementation of *M. truncatula nsp*2-2 using *NSP2* or *HvNSP2* and quantification nodule numbers at 14 dpi (Sm2011, OD600 = 0.01). Data are mean ± SD. Letters denote statistically significant groupings analyzed by one-way ANOVA with Tukey’s test. Experiments were repeated twice with similar results. (D) Representative transgenic roots expressing *M. truncatula NSP2* or *HvNSP2*. (E) Heatmaps showing the expression of a subset of barley *HvMYB* genes in response to N and P starvation. The *Hvnsp* mutants show gene expression in *Hvnsp* mutants compared with wild-type plants under nutrient depletion, while *NSPox* shows *NSP* overexpression in roots compared with wild type under nutrient-replete conditions. The concentrations used were defined as follows: −N−P, no NO^3−^ and no PO_4_^3−^; −N+P, no NO^3−^ and 0.5 mM PO_4_^3−^; +N−P, 5 mM NO^3−^ and no PO_4_^3−^; and +N+P, 5 mM NO^3−^ and 0.5 mM PO_4_^3−^. Color scale represents log2 fold change. Data from Li et al.^48^ See also Figures S5 and S6.

Given that nodule symbiosis evolved from the AM symbiosis^14^ and that flavonoids function in both associations,^21,22^ we hypothesized that NSP2-MYB40 regulation of flavonoids could be important for the interaction with AM fungi. As strigolactones are essential for AM symbiosis and are regulated by *NSP1/NSP2*, we first determined if strigolactone biosynthesis genes were affected in the *myb40* mutants (Figure S5A), as this could be a potential confounder of the mycorrhizal *NSPs* function mediated by flavonoids. This revealed that no change in *D27* and *CCD8* strigolactone biosynthesis genes was detected. We then investigated the potential AM symbiotic phenotype of *M. truncatula MYB40* function using *myb40* mutants inoculated with *Rhizophagus irregularis*. At 35 dpi, R108 roots displayed about 19% root length colonization with arbuscules, indicative of early-stage symbiosis establishment. By contrast, both *myb40* mutant alleles exhibited an ~65% reduction in colonization (Figure 5A). A close examination revealed arbuscule morphology appeared normal in all genotypes, with fully developed structures observed in both the wild type and the mutants (Figure 5B). Together, the observed mutant phenotype points to an impaired early symbiotic signaling between the plant and fungus, while the development of the symbiotic interface is not noticeably affected.

To sustain a role of *NSPs* in AM establishment through the regulation of flavonoid biosynthesis, we examined whether flavonoid biosynthesis genes in barley (*Hordeum vulgare*) are similarly regulated by *HvNSPs*. Consistent with this hypothesis, the induction of many genes, including *HvPAL* and *HvCHS*, under nutrient deficiency (−N−P) was abolished in *Hvnsp1* and *Hvnsp2* mutants, whereas overexpression of *Medicago NSP1* or *NSP2* in barley increased their expression under nutrient-sufficient (+N+P) conditions (Figure S5B). Intriguingly, although barley does not form nodules, *HvNSP2* was able to rescue the nodulation-defective phenotype of the *Mtnsp2-2* mutant (Figures 5C and 5D), in line with previous findings that rice *OsNSP2* complements the *Ljnsp2* mutant.^65^ We further found that several barley *HvMYB* genes were strongly upregulated during nutrient starvation (Figure 5E) and identified a physical interaction between HvNSP2 and HvMYB7 (a homolog of MYB40) in *N. benthamiana* (Figure S5D). These results suggest that an NSP2-MYB module may exist in barley, although its function requires further investigation. As a follow-up, notably using loss-of-function mutants.

Collectively, our results suggest that, as shown in *M. truncatula*, NSP2 may regulate flavonoid biosynthesis to facilitate the establishment of microbial symbiosis under nutrient starvation, potentially by recruiting a MYB40-like homolog.

## Discussion

Mutualistic microbial interactions of plants are primarily associated with the acquisition of nutrients from the surrounding environment.^1,3^ In cases such as the association between legumes and nitrogen-fixing bacteria or that between plants and AM fungi, these microbial associations come with a significant cost to the host plant, principally through the underpinning support of microbial metabolism.^4,15^ These energetic costs provide drivers for the plant to be selective with regard to its engagement with mutualistic microorganisms, most notably regulating these symbioses as a function of the plants’ nutritional needs.^1^ We and others have previously shown that *NSP1/NSP2* link the plants’ nutritional status to the induction of strigolactones,^47–49^ which act as plant-derived rhizospheric signals to AM fungi.^50^ Here, we show that *NSP1/NSP2* also regulate the flavonoid biosynthesis pathway, producing the important rhizospheric signals to nitrogen-fixing bacteria, as well as to AM fungi. This function of *NSP2* requires the action of MYB40, which provides the transcriptional anchor for NSP2 activation of the flavonoid biosynthesis pathway. This fits an emerging picture whereby *NSP2* coordinates multiple stages of nodulation in the legume-rhizobia symbiosis, forming diverse transcriptional complexes that activate different processes associated with the establishment of nitrogen fixation.

The NSP proteins were initially identified because of their essential role in nodulation of legumes,^40,44^ and consistently they were found to transcriptionally activate key components of the nodulation pathway,^41–43^ acting with DELLA and CYCLOPS to form transcriptional complexes that directly bind early nodulin gene promoters.^66,67^ This explained their function in nodule initiation. However, it is becoming increasingly apparent that the *NSP1/NSP2* also act before contact between the host plant and its symbiont,^47–49^ allowing the perception of nutrient limitation to activate production of rhizospheric signals to the mutualistic symbionts in the soil. This is the case for strigolactones to mycorrhizal fungi^48^ and, as shown here, flavonoids to rhizobial bacteria and potentially also to mycorrhizal fungi.

We have recently proposed that *NSP1/NSP2* act as “nutrient checkpoints”: through their regulation by nutrient limitation and their requirement at multiple steps in symbiosis, they provide a means for nutrient availability to restrict or promote the symbiotic process.^52^ By necessitating the action of NSPs at points of plant signaling in the rhizosphere,^48^ symbiosis signaling following LCO perception,^10,43^ and the activation of nodulation,^64,68,69^ the plant places a nutrient checkpoint into the development of nitrogen fixation, such that nodulation only progresses when nitrogen is limiting and remains limiting. From the work here and else-where,^52,61,66,67^ we can infer that NSPs function in different transcriptional complexes at these different points in the nodulation process: NSP2 in complex with MYB40 at the point of flavonoid production, NSP1 and NSP2 in complex with CYCLOPS and DELLAs at the point of symbiosis signaling,^66,67^ and NSP2 possibly in complex with response regulators during the activation of nodule development by cytokinin.^64,68–70^ The evolutionary integration of NSP2 into the action of these transcriptional regulators may directly couple nutrient signaling to the activation of nitrogen-fixing bacteria.

The *NSPs* initially evolved during the regulation of the mycorrhizal symbiosis,^48,52^ and we show that the NSP2-MYB40 module is also relevant for appropriate mycorrhizal colonization, likely as a result of their control of flavonoid biosynthesis in *M. truncatula* and also potentially in barley (Figure 5). We have previously demonstrated that *NSPs* regulate strigolactone biosynthesis as a function of nutrient availability,^48^ and this, along with the present work, highlights the broader role of NSPs during the regulation of a range of secondary metabolites. Our work implies that the NSP2-MYB40 module existed prior to the emergence of nodulation in legumes, and the co-option of NSP2 functionality into the legume-rhizobial symbiosis does not appear to require an additional function of NSP2, since barley *NSP2* can complement a *Medicago nsp2* mutant (Figure 5). This reinforces that *NSP* functionality evolved with the onset of the mycorrhizal association at the base of the plant kingdom^71,72^ and was later recruited into nitrogen fixation in legumes, likely alongside the broader recruitment of mycorrhizal signaling into nodulation.

It is noted that the *myb40* single mutant shows only a partial impairment under rhizobia-limiting conditions (Figures 2 and S3). This is likely due to genetic redundancy with *MYB41*, as evidenced by the stronger nodulation defect in an *MYB41-RNAi/myb40* double mutant. This also suggests that the NSP2-MYB40 module may fine-tune metabolic aspects of the symbiosis, possibly to optimize symbiotic efficiency under suboptimal rhizobial densities.

In addition to the NSP2-MYB40 module, *NSP1* is also required for nutrient regulation of the flavonoid biosynthesis pathway and can autoactivate this pathway, to a degree, when overexpressed (Figure 1). However, in the transactivation assay in *N. benthamiana*, we see principally a requirement for NSP2 action but little evidence to support a role for NSP1 (Figures 1H and 4D). This discrepancy may be attributed to interference from endogenous NSP homologs in *N. benthamiana*. Notably, *NSP2* expression trended lower in the *nsp1* mutant but not reciprocally for *NSP1* in the *nsp2* mutant (Figure S6), and it is possible that, at least at this stage of the symbiosis, the principal function of NSP1 may be the contribution to appropriate *NSP2* induction. Furthermore, a recent study identified NIN as a regulator of isoflavone metabolism in *M. truncatula*,^38^ indicating that *NSP1/NSP2* may also function indirectly in flavonoid modulation partly through NIN. In other contexts, NSP1 appears to act within a protein complex that controls gene expression, a mechanism that requires future elucidation. The emergence of spatiotemporal transcriptomics^73,74^ and cell-type-specific gene editing^75^ will enable future studies to better dissect the genetic relationships between *NSP1, NSP2*, and *MYB40* in regulating flavonoid biosynthesis.

Flavonoids have been demonstrated to act as rhizospheric signals to rhizobial bacteria^25,26,57^ and also to control auxin transport during the activation of cell division in the initiation of the nodule meristem.^29,30^ In *M. truncatula*, cytokinin response 1 (CRE1)-mediated pathway induces flavonoid biosynthesis to regulate auxin transport,^30,68,76^ which is essential for nodule development. Furthermore, CRE1 signaling activates the expression of *NSP2* during nodule organogenesis.^64,68^ Critically, gain-of-function of the cytokinin receptor induces spontaneous nodules requiring *NSP1/NSP2*,^77^ pointing at a late requirement for *NSPs* in the development of the nodule itself. It is therefore possible that the promotion of flavonoid biosynthesis by *NSPs* may be relevant not only at early stages of the interaction but also at the later stage of nodule inception.

One limitation of our study is the lack of a comprehensive flavonoid profile in the *nsp1, nsp2*, and *myb40* mutants. Although we quantified two specific flavonoid compounds in root extracts, this approach may not fully capture their dynamics in the rhizosphere. This is because symbiosis-relevant flavonoids are actively secreted into the soil to attract microbes, making their *in situ* concentrations difficult to capture accurately. Furthermore, the biosynthesis of such flavonoids is often induced in a spatiotemporally specific manner during rhizobial infection. Thus, while overall flavonoid content in root tissues may remain largely unchanged, concentration changes could occur specifically in rhizobia-infected root hairs and nodule primordia.

In variable environments, plants must rapidly sense and respond to nutrient (N/P) availability, a process that involves both local and systemic signaling.^1,4^ Several components, including C-terminally encoded peptides (CEPs)^78,79^ and the NIN-like protein (NLP)/CLAVATA3-like peptide (CLE) pathway,^80,81^ play essential roles in adaptive responses to N status and regulation of nodulation. While the relationship between these established pathways and the NSP2-MYB module identified in our study remains unclear, their coexistence supports the broader concept of nutrient checkpoints that we propose.^52^ A key question is how plants integrate these multiple signals to coordinate development with microbial engagement, thereby optimizing nutrient capture and regulating overall growth. Understanding this network will provide a foundation for engineering crops with better N/P use efficiency and climate resilience.

Our work shows a new mode of action of NSP2: by binding MYB40, it controls the expression of the flavonoid biosynthesis pathway as a function of nitrogen and phosphorus availability. The NSP2-MYB40 module acts in the promotion of both the nitrogen-fixing rhizobial symbiosis and the mycorrhizal symbiosis. We propose that the requirement for *NSP2* action at this stage in both symbiotic associations allows nutrient perception to control the communication with these beneficial microorganisms, enabling a “nutrient checkpoint” to control microbial signaling.

## Resource Availability

### Lead contact

Requests for further information and resources should be directed to and will be fulfilled by the lead contact, Jin-Peng Gao (jg2133@cam.ac.uk), subject to material transfer agreements.

### Materials availability

Plant materials used in this study are available from the lead contact upon request.

## Star★Methods

### Key Resources Table

**Table:** 

REAGENT or RESOURCE	SOURCE	IDENTIFIER
Antibodies		
anti-FLAG	Insight Biotechnology	SAB-48045; RRID: AB_3713276
anti-GFP	Insight Biotechnology	SC-9996-HRP; RRID: AB_3713277
anti-mCherry	Origene	SKU-TA183007; RRID: AB_3731276
Bacterial and virus strains		
*Sinorhizobium meliloti* 2011	Lab stock	N/A
*Sinorhizobium meliloti* 2011 pXLGD4 lacZ	Lab stock	N/A
*Agrobacterium rhizogenes* Arqua1	Lab stock	N/A
*Agrobacterium tumefaciens* GV3101	Lab stock	N/A
*Escherichia coli* DH5α	Lab stock	N/A
*Escherichia coli* BL21(DE3)	New England Biolabs	Cat#C2527H
Chemicals, peptides, and recombinant proteins		
5-Bromo-4-chloro-3-indolyl-β-D-glucuronic Acid (X-gluc)	Melford	15548-60-4
5-Bromo-β-chloro-3-indolyl-β-D-galactopyranoside (X-Gal)	Sigma-Aldrich	7240-90-6
isoliquiritigenin	Fluorochem	F462708
naringenin	Cambridge Bioscience	T2838
Proteinase inhibitor cocktail	APExBIO	K1011-10
isopropylthio-β-galactoside (IPTG)	Melford	367-93-1
Precast polyacrylamide gel	Bio-Rad	4565013
Yeast media SD/-Trp/-Leu	Takara	630317
Yeast media SD/-Trp/-Leu/-His	Takara	630319
Critical commercial assays		
Gibson Assembly Master Mix	New England Biolabs	E2611S
Luciferase Assay System	Promega	E1500
RNeasy Plant Mini Kit	Qiagen	74904
One-Step gDNA Removal and cDNA Synthesis Super Mix	TransGen	AT311-03
Luna Universal qPCR Master Mix	New England Biolabs	M3003L
Dual-Luciferase Reporter Assay System	Promega	E1910
Plant ChIP Kit	Epigentek	P-2014
Deposited data		
Raw RNA-seq data	This study	PRJNA1277521
Experimental models: Organisms/strains		
*Medicago truncatula* A17	Lab stock	A17
*Medicago truncatula* R108	Lab stock	R108
*Medicago truncatula nsp*1-1	Catoira et al.^44^	*nsp1-1*
*Medicago truncatula nsp*2-2	Oldroyd and Long^40^	*nsp2-2*
*Medicago truncatula myb40-1*	Oklahoma State University	NF21386
*Medicago truncatula myb40-2*	Oklahoma State University	NF9316
*Rhizophagus irregularis*	Lab stock	N/A
*Nicotiana benthamiana*	Lab stock	N/A
Yeast strain Y2HGold	Takara	Cat#630498
Oligonucleotides		
Primers	Table S1	N/A
Recombinant DNA		
Golden Gate plasmids	GeneArt, Thermo Fisher Scientific	https://www.ensa.ac.uk
pGBKT7-MYB40	Clontech	N/A
pGEX-4T-1	GE Healthcare	Cat#28-9545-49
Software and algorithms		
ImageJ	https://fiji.sc	N/A
GraphPad Prism 7	https://www.graphpad.com/	Prism 7
PhyML	https://ngphylogeny.fr/	N/A
MEME	https://meme-suite.org/meme/	N/A
UCSF ChimeraX	https://www.rbvi.ucsf.edu/chimerax/	N/A
Detailed analysis scripts	This study	https://github.com/chongjing/RNAseq_Medicago
Trimmomatic	Bolger et al.^82^	v0.39
Novoalign	http://www.novocraft.com	v4.03.08
HTseq-count	Putri et al.^83^	HTSeq 2.0
EdgeR	Robinson et al.^84^	N/A

### Experimental Model and Subject Details

#### Plant Materials and Growth Conditions

*Medicago truncatula* ecotypes A17 and R108 were used as wild-type in this study, dependent on the ecotype of the respective mutant. The mutants of *nsp1-1* and *nsp2-2* (both are Jemalong A17 background) were reported previously.^40,44^ The Tnt1 retrotransposon insertion lines used in this study are in the R108 genetic background. These include: NF21386 (*myb40-1*) and NF9316 (*myb40-2*). Both mutant lines were obtained from the *Medicago truncatula* Mutant Database^85^ (Oklahoma State University, Stillwater, USA). *Nicotiana benthamiana* was used for transient transformation assays.

*M. truncatula* seeds were scarified, surface sterilized with 10% (v/v) bleach solution, stratified for 2 days at 4°C and germinated on water agar plates at 22°C. For standard plant growth, the seedlings were grown in a mixed soil containing a 3:1 ratio of peat soil and vermiculite unless otherwise stated. Plants were watered twice weekly and maintained in controlled environment chambers with 16 h of light, 8 h of dark photoperiod at 22°C with 55% relative humidity, and the light intensity of 150 μmol m^-2^ s^-1^.

#### Microbial Strains

The *rhizobium Sinorhizobium meliloti* 2011 (Sm2011) expressing pXLGD4 (*hemA: lacZ*) was used in this study for nodulation assays. The model arbuscular mycorrhizal fungal species, *Rhizophagus irregularis* was used in this study for mycorrhizal inoculation. *Agrobacterium rhizogenes* Arqua1 strain was used for hairy root transformation. The strain *A. tumefaciens* GV3101 was used for transient transformation in *N. benthamiana*. Yeast strain Y2HGold (630498, Takara, Kusatsu, Shiga, Japan) was used for yeast two hybrid. For gene cloning and protein expression, *Escherichia coli* DH5α and BL21 (DE3) strains were used, respectively.

### Method Details

#### Vector Construction

The gene and promoter sequences in this study were commercially synthesized (GeneArt, Thermo Fisher Scientific, Waltham, MA, USA), and subsequently cloned into destination vectors by Golden Gate assembly^86^ or Gibson assembly (E2611S, NEB, Ipswich, MA, USA), as detailed in the following methods. For RNAi analysis, two different target regions of *MYB41* were designed with the pssRNAit web server.^87^ The fragments MYB41-RNAi-1 (20/207 bp from ATG) and MYB41-RNAi-2 (672/864 bp) were synthesized and cloned into the modified vector pK7GWIWGIIR.^66^ The primers used in this study are listed in Table S1. All the Golden Gate Level 0 vectors are available through the ENSA project core collection (https://www.ensa.ac.uk/).

#### Gene Expression Analysis

The RNA-seq data of *M. truncatula* and barley (*Hordeum vulgare*) under different nutrient conditions were obtained from a previous study.^48^ The gene expression data for spot inoculation of rhizobia in *M. truncatula* were sourced from an earlier work.^54^ The heatmaps were generated using GraphPad Prism 7 (GraphPad Software, San Diego, CA). For RNA-seq of *nsp2-2* mutant after rhizobial inoculation (Sm2011, OD600=0.1), whole roots from Fahraeus plant (FP) agar plates were collected at 7 days post inoculation (dpi). Total RNA was extracted with the RNeasy Plant Mini Kit (74904, Qiagen, Valencia, CA, USA), following the manufacturer’s instructions. The sequencing of the libraries was performed by Novogene Europe (Cambridge, UK) with 150 bp paired-end reads. Raw RNA-seq data have been deposited in the NCBI database under accession number BioProject PRJNA1277521. For RNA-seq analysis of *nsp2-2* mutant, *M. truncatula* v4.0 genome and associated annotation (https://phytozome-next.jgi.doe.gov/info/Mtruncatula_Mt4_0v1) were used as reference. Raw reads were filtered and trimmed to get high quality reads using Trimmomatic v0.39.^82^ Briefly, the bases with quality less than 20 at the start or end of a read were cut off, and reads with length shorter than 60 bp were dropped. Novoalign (v4.03.08; http://www.novocraft.com) was used to align clean reads to reference genome, and expression counts were calculated using HTseq-count.^83^ Differential expression analysis was performed using edgeR,^84^ and the differentially expressed genes are presented in Data S1 and S2.

For quantitative real-time PCR (qPCR), 1–2 μg of total RNA was reverse-transcribed into cDNA using the One-Step gDNA Removal and cDNA Synthesis Super Mix (AT311-03, TransGen, Beijing, China). qPCR was performed using Luna Universal qPCR Master Mix (M3003L, NEB, Ipswich, MA, USA) with the real-time PCR detection system (CFX96, Bio-Rad, Hercules, CA, USA). The PCR conditions were as follows: 40 cycles of 95°C for 15s, 60°C for 15s, and 72°C for 15s. The primers used for gene expression analysis are listed in Table S1.

#### Hairy Root Transformation

The hairy root transformation assay was performed as described.^88^ Briefly, *M. truncatula* seedlings were cut slantwise above the hypocotyl on a sterile flow bench, and then the wounded area was dipped in a culture of *A. rhizogenes* Arqua1 transformed with a given binary vector. The seedlings were then placed on Fahraeus plant (FP) medium for 1 week at 22°C. The newly grown roots of these seedlings were cut again and then transferred to modified FP medium containing 0.5 mM KNO_3_ for 3 weeks. The positive transgenic roots were identified using an anthocyanin-based visual marker.^89^ Following the removal of non-transgenic roots, the transgenic composite plants were then used for rhizobial inoculation.

#### Histochemical GUS Staining

For promoter-GUS analysis in *M. truncatula* transgenic roots, 2000 bp promoter fragments of *IMaT7* and *CYP75B1*, 1200 bp of *ChOMT1*, and 3000 bp of *MYB40* were cloned into the pCAMBIA1381 vector, respectively, by recombination reactions (E2611S, NEB, Ipswich, MA, USA). These vectors were then introduced into *A. rhizogenes* strain Arqua1 for hairy root transformation. The transgenic roots were harvested and washed twice times by 0.1 M sodium phosphate buffer (pH=7.0) and incubated in a GUS staining solution comprising 0.5 mg/mL 5-bromo-4-chloro-3-indolyl-β-D-glucuronic acid (X-gluc, 15548-60-4, Melford, Ipswich, Suffolk, UK), 0.5 mM potassium ferricyanide, 0.5 mM potassium ferrocyanide, 0.1% (v/v) Triton X-100, and 0.1 M sodium phosphate buffer at 37°C in the dark for 6–12 h. The roots were rinsed washed with 70% ethanol (v/v) three times, and the samples were imaged with a stereo microscope (S9D, Leica, Wetzlar, Germany) and a widefield microscope (DM750, Leica, Wetzlar, Germany).

#### Nodulation Assay

Wild type, stable mutants, and transgenic composite *M. truncatula* plants were grown in 5 × 5 × 5.5 cm^3^ pots containing 1:1 mix of sterile terra green (Oil-Dri UK Ltd) and sand. After 7-10 days of growth, plants were inoculated with 1 mL of Sm2011 suspension at OD600 of 0.1 or 0.01 per pot, as indicated in the article. At 5 days and 14 days post inoculation (dpi), roots were harvested and nodules were scored. To detect infection threads, the roots were histochemically stained in 0.1 M sodium phosphate buffer (pH=7.4) containing 0.8 mg/mL 5-bromo-4-chloro-3-indolyl-β-D-galactopyranoside (X-Gal, 7240-90-6, Sigma-Aldrich, Merck KGaA, Darmstadt, Germany), 10 mM KCl and 1 mM MgSO_4_ at 28°C in dark overnight to stain the lacZ-tagged rhizobia. Infection threads were then scored under a widefield microscope (DM750, Leica, Wetzlar, Germany).

#### Mycorrhizal Inoculation and Assessment

*M. truncatula* wild-type and mutant plants were grown in a modified container made from 50 mL falcon tubes with a drainage hole. To prevent light exposure to roots, the tubes were wrapped in black tape. Inoculation followed previous work.^90^ Seedlings with approximately 5 cm root system were planted in a sand-terra green substrate (9:1 ratio), with each cone hosting two seedlings and 300 *R. irregularis* spores. Seedlings were watered with reverse osmosis (RO) water for the first week, followed by an alternating regime of Hoagland’s solution (25 μM Pi) and RO water. Roots were harvested at the specified time points and stained with 0.05% (w/v) trypan blue. At harvest, root systems were cut into 1 cm fragments and divided up for staining. Assessment of root length colonization was performed with a modified gridline intersect method described previously^90,91^ at 20X magnification objective using DM750 Microscope (Leica, Wetzlar, Germany) and expressed as percentage of the total root length scored. Representative images were taken using GXML2800 microscope (GT Vision, Stanfield, UK).

#### Flavonoid Treatments

For exogenous flavonoid applications, 3 μM flavonoids were used, as previous studies^25,30^ showed that this concentration could restore nodulation in flavonoid-deficient roots. The plant roots were treatment into the solution containing an equimolar mixture of isoliquiritigenin (F462708, Fluorochem, Hadfield, Derbyshire, UK), and naringenin (T2838, Cambridge Bioscience, UK). Control treatments contained equivalent dilutions of ethanol used as a solvent for stock solutions. Plants were inoculated with Sm2011 at OD600=0.1 or OD600=0.01, as indicated. Roots were harvested at 14 days post inoculation for nodule quantification.

#### Extraction and Quantification of Flavonoids

Extraction of flavonoids from roots was performed based on a previous report.^26^ Briefly, the roots were carefully collected after three days post-inoculation. The roots were flash-frozen in liquid nitrogen and ground to a fine powder. 100 mg of powdered tissue was extracted with 80% methanol (v/w, 5:1) at 4°C. The mixture was centrifuged three times at 13,500 g for 10 min to remove precipitates and the supernatant was collected. Six biological replicates were included per sample.

Following extraction, flavonoids were analyzed by a QTRAP 6500+ LC-MS/MS system (SCIEX, Framingham, MA, USA). The mass spectrometer was coupled to an Acquity UHPLC system (Waters, Milford, MA, USA) equipped with a Poroshell 120 SB-Aq column (100 × 3.0 mm, 2.7 μm; Agilent, Santa Clara, CA, USA). The mobile phase A was 2 mM ammonium formate and 0.1% formic acid in water and B was methanol. The column was maintained at 40°C with a flow rate of 0.4 mL min^-1^, and the gradient of B was as follows: 0 min, 10%; 1.5 min, 45%; 9 min, 55%; 10 min, 95%; 12 min, 95%; 12.1 min, 10%; 15 min, 10%. All analytes were detected using multiple reaction monitoring (MRM) mode. The optimized ESI operating parameters for negative mode were: ion spray voltage, -4.5 kV; ion spray temperature, 500°C; curtain gas, 35 psi; ion source gas 1, 50 psi; ion source gas 2, 50 psi.

#### Yeast Two Hybrid Assay

The coding region sequence (CDS) of *MYB40* was inserted into pGBKT7 by recombination reactions (E2611S, NEB, Ipswich, MA, USA). The constructs pGADT7-NSP1 and pGADT7-NSP2 were generated in a previous study.^43^ Different pairs of constructs were introduced into yeast strain Y2HGold (630498, Takara, Kusatsu, Shiga, Japan), according to the manufacturer’s protocol. The cells were grown on yeast minimal media/synthetic-defined (SD)-Trp/-Leu (630317, Takara, Kusatsu, Shiga, Japan), SD-Trp/-Leu/-His (630319, Takara, Kusatsu, Shiga, Japan), and SD-Ade/-His/-Leu/-Trp (630323, Takara, Kusatsu, Shiga, Japan). Yeast growth was monitored for 3 to 5 days, and the interactions were analyzed based on yeast growth on the selection media.

#### Spilt Luciferase Complementation Assay

The *MYB40* was cloned into a modified p35S-nLUC vector^92^ and *NSP2* was cloned into a modified p35S-cLUC vector^92^ by recombination reactions (E2611S, NEB, Ipswich, MA, USA). The resulting plasmids were transformed into *A. tumefaciens* GV3101 respectively, and then cultivated overnight to OD600= 1.0. The cultures were collected and resuspended in infiltration buffer comprising 10 mM MgCl_2_, 10 mM MES, and 250 mM acetosyringone. Different pairs of solution were mixed and incubated at room temperature for 2 hours, then co-injected into *N. benthamiana* leaves with *A. tumefaciens* carrying *P19* for transient expression. After 3 days of growth, the leaves were sprayed with 1 mM luciferin (E1500, Promega, Madison, WI, USA) and the luminescence was detected and photographed using the ImageQuant 800 system (GE Healthcare, Chicago, IL, USA).

#### Co-immunoprecipitation

The *M. truncatula* transgenic roots were powdered in liquid nitrogen and total protein was extracted using ice-cold protein extraction buffer comprising 50 mM Tris-HCl (pH 7.5), 150 mM NaCl, 10% glycerol (v/v), 1 mM dithiothreitol (DTT), 1 mM EDTA, 0.5% Triton-X100 (v/v) and proteinase inhibitor cocktail (K1011-10, APExBIO, Houston, TX, USA). The proteins were then incubated with prewashed anti-FLAG M2 magnetic beads (A2220, Sigma-Aldrich, Merck KGaA, Darmstadt, Germany) for 2–6 h at 4°C on a roller shaker. The beads were collected at 4°C, 500 rpm for 2 min and washed 3 to 5 times with extraction buffer. The agarose beads with buffer were added in 4×SDS loading buffer and boiled for 5min. The resulting samples were separated on 4-20% precast TGX gel (456-1093, Bio-Rad, Hercules, CA, USA). The proteins were detected by western blot analysis using anti-FLAG (SAB-48045, Insight Biotechnology, Wembley, UK) and anti-GFP (SC-9996-HRP, Insight Biotechnology, Wembley, UK) and the bands were detected using the ImageQuant 800 system (GE Healthcare, Chicago, IL, USA).

#### Subcellular Localization

The CDS of *NSP2* was fused in-frame to the green fluorescent protein (GFP) and driven by *LjUBQ* promoter to construct the *pLjUBQ:GFP-NSP2-tHSP* vector using Golden Gate assembly.^86^ For co-localization of *NSP2* and *MYB40* in *N. benthamiana*, a construct containing *pLjUBQ:GFP-NSP2-tHSP* and *pZmUBQ:MYB40-mCherry-tNOS* was generated by Golden Gate assembly. The construct was transformed into *A. tumefaciens* GV3101 and infiltrated into *N. benthamiana* leaves, then analyzed by confocal microscopy (Stellaris 8 Falcon, Leica, Wetzlar, Germany) after 3 days of growth. Fluorescence was detected using excitation wave-lengths of 488 nm (GFP) and 561 nm (mCherry), with emission collected at 500-530 nm (GFP) and 600-630 nm (mCherry).

#### Structure Prediction and Analysis

Three-dimensional structures of the MYB40-DNA complex, MtNSP2, and HvNSP2 were predicted using AlphaFold 3 with default parameters. For each protein, the model with the highest pLDDT score and pTM score was selected for subsequent analysis. Structural alignment and figure preparation were performed in UCSF ChimeraX2.^93^

#### Protein Expression and Electrophoretic Mobility Shift Assay

The CDS of *NSP1, NSP2*, and *MYB40* were cloned into pGEX-4T-1 vector and in-frame with Glutathione-S-transferase (GST) by recombination reactions (E2611S, NEB, Ipswich, MA, USA). Plasmids were transformed into the *E. coli* strain BL21 (DE3) for expression as GST-tagged fusion proteins. Expression of the fusion proteins was induced with 0.5 mM isopropylthio-β-galactoside (IPTG, 367-93-1, Melford) for 8–12 h at 28°C. The bacterial cultures were centrifuged at 4°C to pellet the cells and subsequently resuspended in ice-cold phosphate buffer (pH=7.4). Cell lysis was then performed through ultrasonication using a sonicator (Q500, Qsonica, Newtown, CT, USA). Electrophoretic mobility shift assay was performed using the recombinant protein and different probes labeled with Cyanine5 (Cy5). The promoter regions of *PAL1* (-292/-244 bp), *CHS1* (-147/-99 bp), *ChOMT1-p1* (-988/-940 bp), *ChOMT1-p2* (-801/-753 bp), *ChOMT1-p3* (-518/-470 bp), *ChOMT1-p4* (-143/-95 bp), *ChOMT1-p5* (-80/-32 bp), *CYP75B1* (-61/-13 bp), and *IMaT7* (-358/-310 bp) were fused with universal adapters to generate specific probes. Unlabeled probes were used as competition probes, and probes with mutations in the binding site were used as mutant competition probes. The protein and probe mixture were incubated at 37°C for 30 min and then electrophoresed on a 5% precast polyacrylamide gel (4565013, Bio-Rad, Hercules, CA, USA) in pre-chilled running buffer under light-protected conditions. The Cy5 fluorescence was detected using the ImageQuant 800 imaging system (GE Healthcare, Chicago, IL, USA).

#### Dual Luciferase Reporter Assay

The promoter fragments of flavonoid biosynthetic genes were cloned into the pGreenII-0800 vector by recombination reactions (E2611S, NEB, Ipswich, MA, USA). The 956 bp promoter fragment of *PAL1*, 1109 bp of *CHS1*, 969 bp of *CHS2*, 1200 bp of *ChOMT1*, 984 bp of *OMT4*, 946 bp of *CYP75B1*, 1000 bp of *IMat7*, and 988 bp of *FLS* were used in the study. These constructs were transformed into GV3101 and then co-injected into *N. benthamiana* leaves with *pLjUBQ:NSP1-mCherry, pLjUBQ:GFP-NSP2* and *pLjUBQ:MYB40-Flag*. After 36 h, three leaves were pooled to constitute one biological replicate. The samples were analyzed using a dual-luciferase reporter assay kit (E1910, Promega, Madison, WI, USA), according to the manufacturer’s protocol. The activities of firefly luciferase and renilla luciferase were measured using a microplate reader (CLARIOstar, BMG Labtech, Ortenberg, Germany).

#### Luminescence Imaging of Transgenic Roots

Bioreporter luminescence in *M. truncatula* transgenic roots was analyzed using the Bright-Glo luciferase assay system (E2610, Promega, Madison, WI, USA) according to previously described methods.^26,69^ Briefly, the 1200 bp promoter fragment of *ChOMT1* was used to drive the expression of *Luciferase* (*LUC*), and the *pChOMT1:LUC* vector was constructed via Golden Gate assembly and then introduced into Arqua1. After hairy root transformation, the plants grown on the FP agar plates were inoculated with either Sm2011 (OD600=0.01) or mock-treated with water. After 2 days post-inoculation, D-luciferin potassium salt (E2610, Promega, Madison, WI, USA) was sprayed onto the roots, and luminescence signals were captured using the ImageQuant 800 system (GE Healthcare, Chicago, IL, USA). To quantify bioluminescent activity, the images were analyzed using ImageJ (https://fiji.sc).

#### Chromatin Immunoprecipitation

The chromatin immunoprecipitation (ChIP) assay was performed using a Plant ChIP Kit (P-2014, Epigentek, Farmingdale, NY, USA). Briefly, 2 g transgenic roots were cross-linked in 20 mL 1% formaldehyde for 5 min under a vacuum. The reaction was quenched with 2.5 mL 1 M glycine. The roots were then ground to a fine power in liquid nitrogen and nuclei were isolated through Miracloth (475855–1R, Millipore, Burlington, MA, USA). The chromatin was sheared using a sonicator (Q500, Qsonica, Newtown, CT, USA). Following immunoprecipitation with an anti-FLAG antibody (SAB-48045, Insight Biotechnology, Wembley, UK), the protein-DNA complexes were reversed cross-linked, and the DNA was purified for qPCR analysis.

### Quantification and Statistical Analysis

Statistical analyses were implemented in GraphPad Prism 7 (GraphPad Software, San Diego, CA). Means were compared using two-tailed Student’s *t* test. Inter-group significance was used one-way ANOVA with Tukey’s test. Samples size (n) and *P*-values are indicated on the figure. Statistical tests are provided in the figure legends.

## Supplementary Material

Supplemental information can be found online at https://doi.org/10.1016/j.cub.2026.01.013.

Data S1

Data S2

Document S1

Document S2

## Data Availability

The genetic materials used in this study are available from the corresponding authors upon request. The raw RNA-seq data have been deposited in the NCBI Sequence Read Archive database under BioProject accession PRJNA1277521. Detailed analysis scripts are publicly available on GitHub at https://github.com/chongjing/RNAseq_Medicago.

## In brief

Gao et al. reveal that an NSP2-MYB40 regulatory module orchestrates flavonoid biosynthesis and symbiotic signaling to dynamically optimize nitrogen acquisition.

## Highlights

- NSP2 activates flavonoid biosynthesis under nutrient starvation
- MYB40 directly regulates flavonoid biosynthetic genes
- NSP2 interacts with MYB40 to enhance nodulation efficiency
- The NSP2-MYB40 module is involved in the mycorrhizal symbiosis
