# Supplementary material for: An NSP2-MYB module orchestrates flavonoid biosynthesis and nodule symbiosis
Source: Curr Biol. Author manuscript; Available in PMC 2026 May 7. (PMC7619063; doi:10.1016/j.cub.2026.01.013)
Supplement: Document S1 [file EMS213625-supplement-Document_S1.pdf]

Current Biology, Volume *36*

Supplemental Information

An NSP2-MYB module orchestrates flavonoid biosynthesis and nodule symbiosis

Jin-Peng Gao (高锦鹏), Chongjing Xia (夏崇靖), Chai Hao Chiu, Qingchao Chen (陈庆超), Suyu Jiang (姜苏育), Xiaotian Wu (吴晓天), Wenjie Liang (梁文杰), Jongho Sun, Min-Yao Jhu, Jiangqi Wen, Ertao Wang (王二涛), Jeremy D. Murray, and Giles E. D. Oldroyd

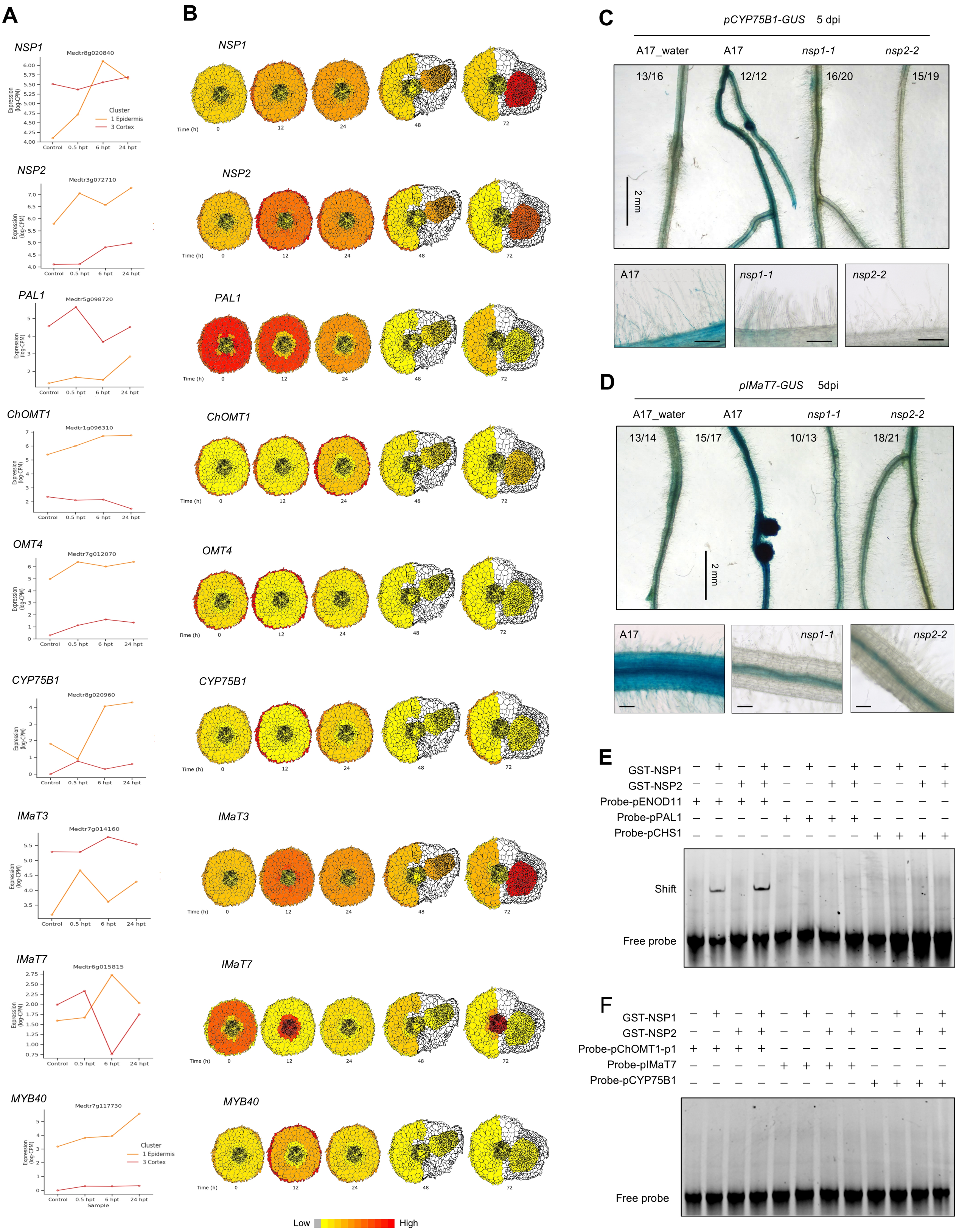

Figure S1. A subset of flavonoid biosynthesis genes exhibits co-expression with *NSP1/NSP2* during nodulation. Related to Figure 1.

(**A**) Genes expression detected in the single-nucleus transcriptomes. Data form Atlas of medicago root[S1] (http://119.45.35.29:3571/).

(**B**) Spatiotemporal expression graphs of *NSP1/NSP2* and the flavonoid biosynthesis genes during nodulation generated at ePlants (https://bar.utoronto.ca/eplant_medicago/).

(**C** and **D**) Promoter activity of *CYP75B1* (**C**) and *IMaT7* (**D**) visualized by GUS in wild-type A17, *nsp1-1*, and *nsp2-2* mutants at 5 dpi. Numbers in the images indicate the numbers of roots having a pattern similar to the one shown in the figure as representative among the total number of stained roots. Images in the lower panels show magnified views, and scale bars correspond to 200 μm.

(**E** and **F**) Electrophoretic mobility shift assays were performed using GST-tagged NSP1 and NSP2 proteins with various Cy5-labeled DNA probes. The promoter regions of *PAL1* (-292/-244 bp), *CHS1* (-147/-99 bp), *ChOMT1-p1* (-988/-940 bp), *CYP75B1* (-61/-13 bp) and *IMaT7* (-358/-310 bp) were used for the assays. The promoter of *ENOD11* was used as a positive control. No specific protein-DNA interactions between NSP1/NSP2 and these flavonoid-related gene promoters were detected under the experimental conditions tested. Experiments were carried out three times with similar results.

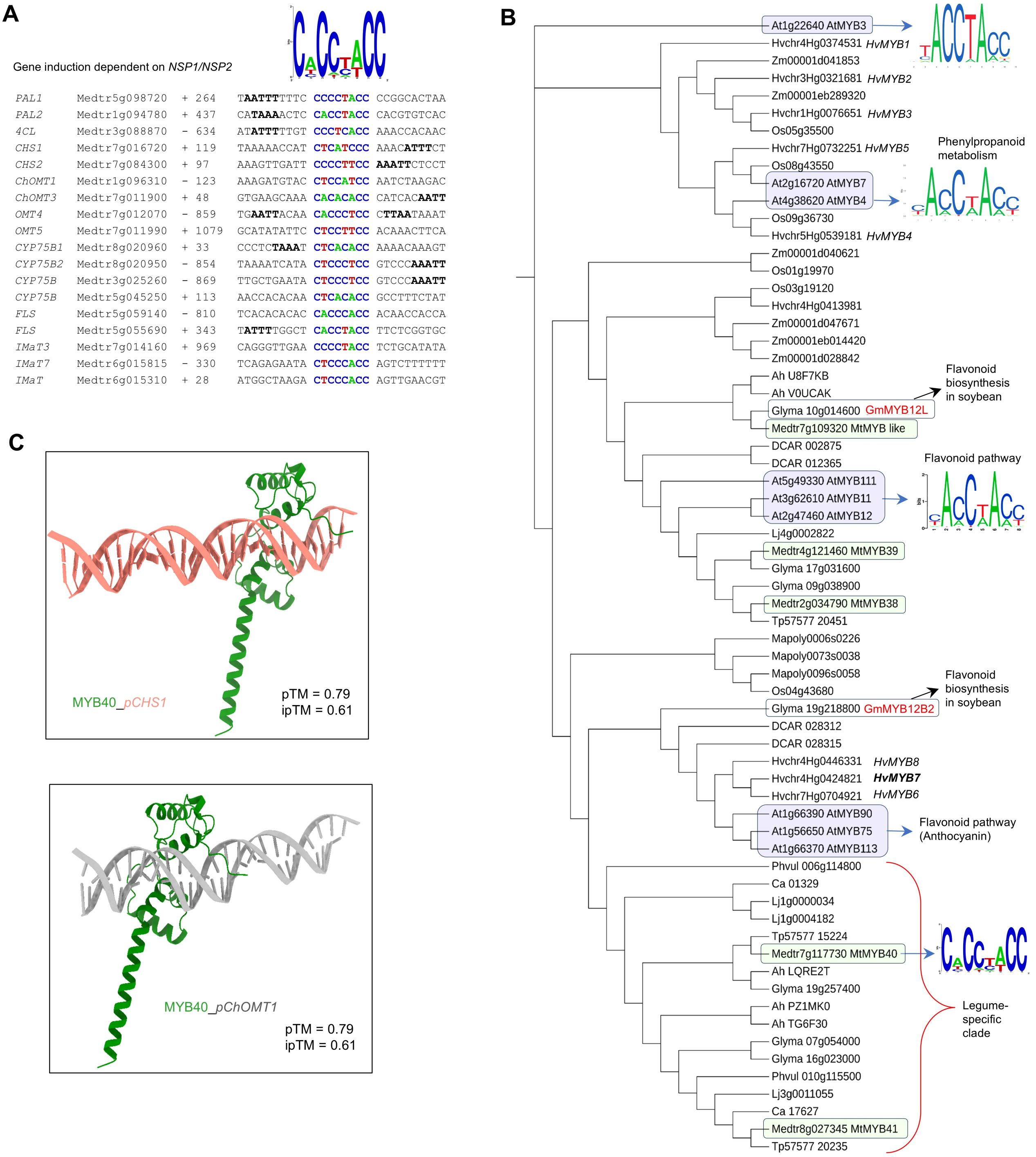

Figure S2. MYB40 is a homolog of known flavonoid regulators. Related to Figure 2 and Figure 4.

(**A**) Analysis of flavonoid biosynthesis gene promoters identified consensus sequences containing a potential MYB-binding element using MEME (Multiple EM for Motif Elicitation).

(**B**) Phylogentic trees represent the evolutionary relationship among a subfamily of flavonoid-regulating MYB transcription factors. The predicted binding motifs for *Arabidopsis thaliana* MYBs were derived from the JASPAR database (https://jaspar2020.genereg.net/).

Amino acid sequences of MYB proteins from *Medicago truncatula*, *Lotus japonicus*, *Glycine max*, *Cicer arietinum*, *Phaseolus vulgaris*, *Trifolium pratense*, *Arachis hypogaea*, *Arabidopsis thaliana*, *Marchantia polymorpha*, *Oryza sativa*, *Zea mays*, *Daucus carota*, and *Fragaria vesca* were obtained from Phytozome database (https://phytozome-next.jgi.doe.gov/). The *Hordeum vulgare* sequence was obtained Golden Promise reference genome (https://ics.hutton.ac.uk/gmapper/index.html). The phylogenetic tree was constructed using PhyML and presented using iTOL (https://ngphylogeny.fr/).
(**C**) Predicted MYB40 and DNA complex structure by AlphaFold3 analysis revealed that the MYB DNA-binding domain directly interacts with flavonoid biosynthesis gene promoters. The promoter fragments of *CHS1* and *ChOMT1* were analyzed as examples. The predicted template modeling (pTM) and interface pTM (ipTM) scores are indicated in the figure.

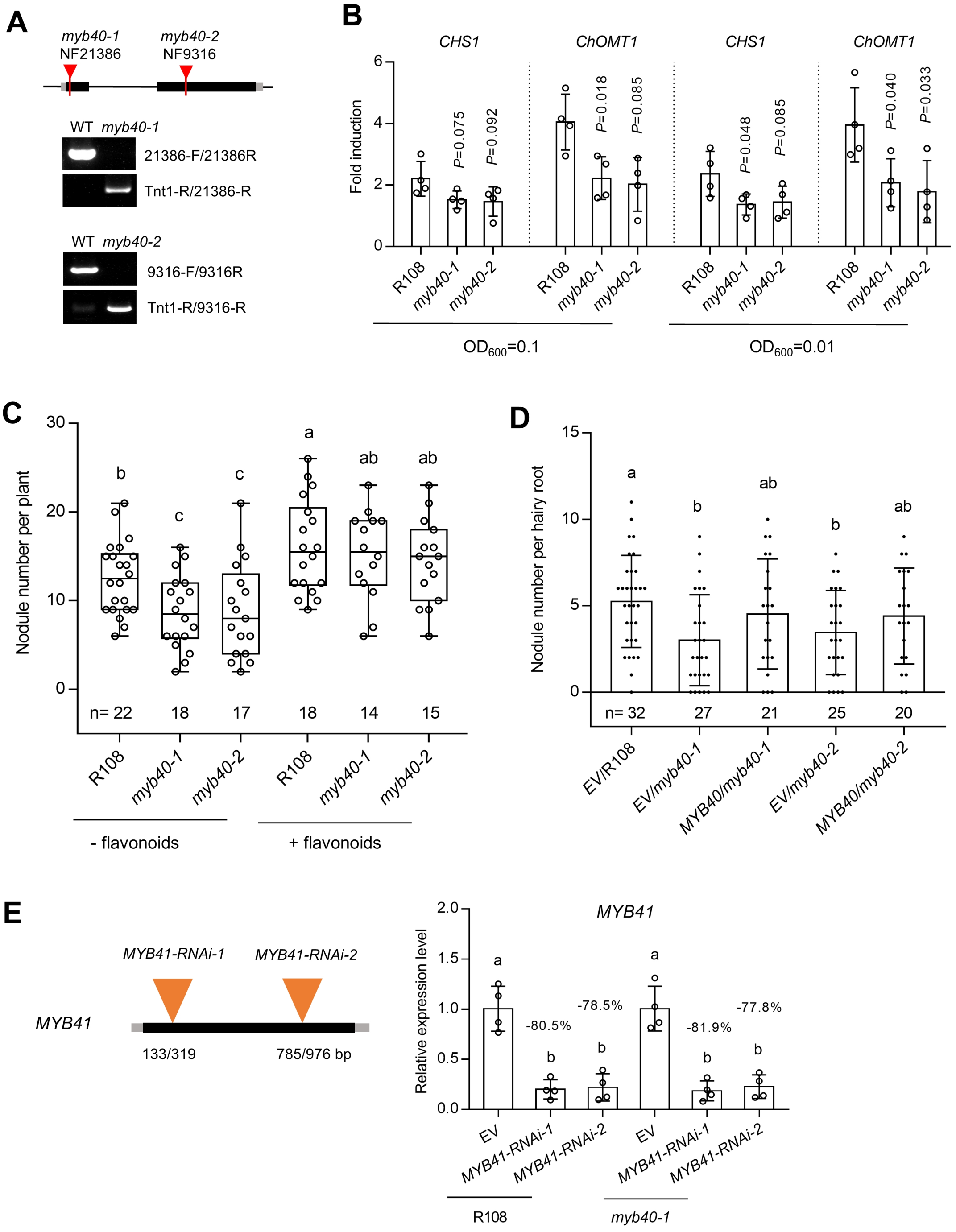

Figure S3. MYB40 is involved in nodulation. Related to Figure 2.

(**A**) Schematic representation of *MYB40* genomic sequence and identification of *Tnt1* insertion mutants of *myb40*. Exons are shown as black boxes. Localizations of *Tnt1* insertion are represented as red lines with red triangles.

(**B**) Expression analysis of *CHS1* and *ChOMT1* in wild-type R108 and *myb40* mutant plants following inoculation with different concentrations of rhizobia. Gene expression levels were normalized against the reference gene *Elongation Factor 1*. Data are mean ± SD. Significant differences were determined by two-tailed Student's *t*-test.

(**C**) Quantification of total nodules at 14 dpi of Sm2011 at OD600=0.01. The three bar graphs on the right show nodulation after flavonoids treatment. Boxes show the first quartile, median, and third quartile; whiskers show minimum and maximum values; dots show data points. Statistical significance was determined by one-way ANOVA with Tukey's test. Experiments were repeated three times with similar results.

(**D**) Genetic complementation of *myb40-1* and *myb40-2* using a full-length *MYB40* coding sequence and quantification of nodule numbers at 14 dpi (Sm2011, OD600=0.01). Numbers below columns represent the number of biologically independent sample sizes. Data are mean ± SD. Means were compared using Student's *t* test. Experiments were repeated twice with similar results.

(**E**) Transcript levels of *MYB41* in empty vector (EV) and *MYB41-RNAi* transgenic roots. Left panel shows two RNAi constructs targeting *MYB41*. Right panel shows *MYB41* transcript levels were reduced by approximately 80% in transgenic roots compared to the EV control. Statistical significance was determined by one-way ANOVA with Tukey's test.

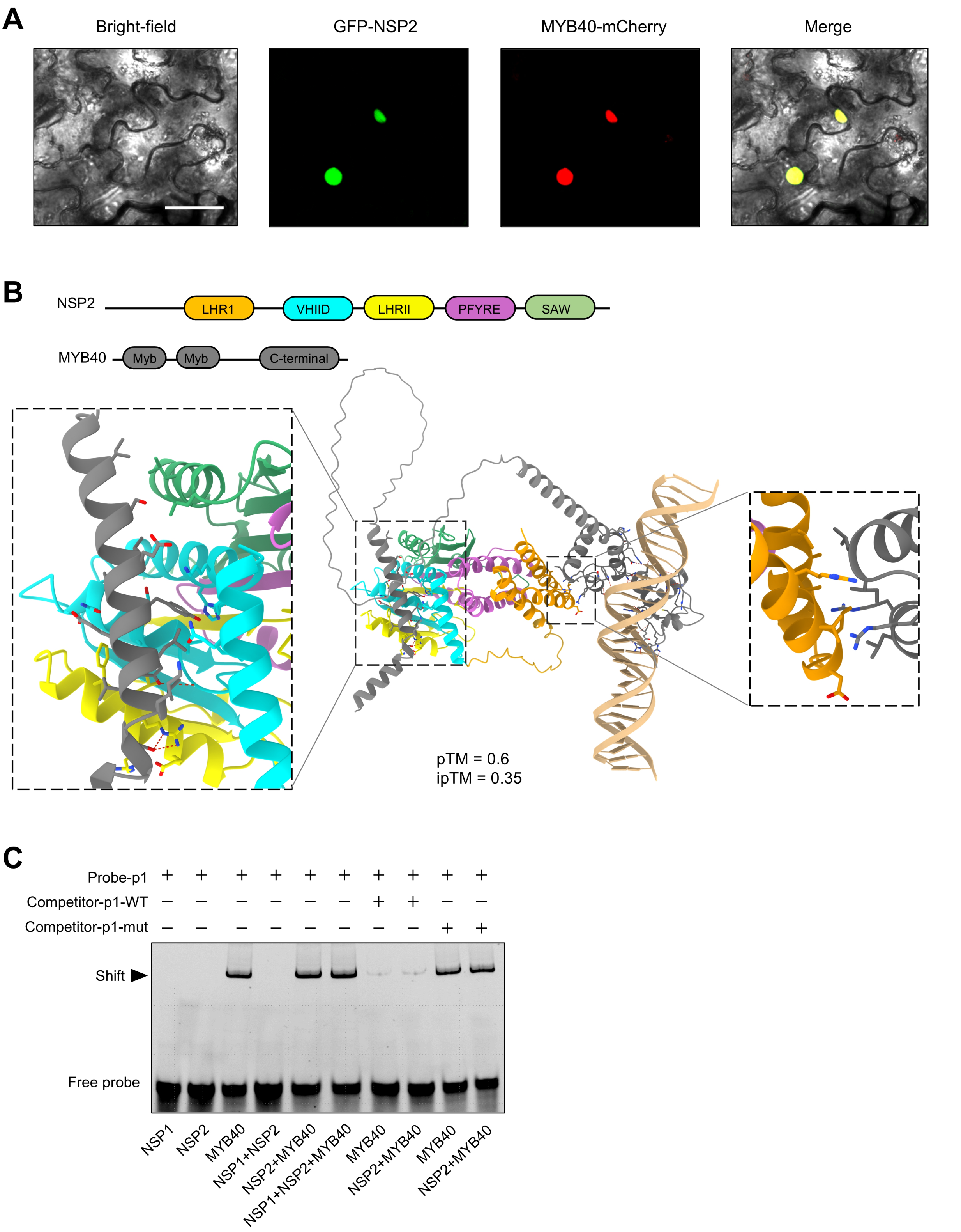

Figure S4. The interaction between NSP2 and MYB40. Related to Figure 3 and Figure 4.

(A) Subcellular localization of GFP-NSP2 and MYB40-mCherry fusion proteins in *N. benthamiana* leaf epidermal cells. *pLjUBQ:*GFP-NSP2 and *pZmUBQ:*MYB40-mCherry showed nuclear co-localization. Scale bar, 50 μm.

(**B**) Interaction prediction between NSP2 and MYB40 by AlphaFold3. DNA is positioned against the MYB40 N-terminal Myb DNA-binding domain, while the VHIID, LHRII, and SAW domains of NSP2 maintain contact with the MYB40 C-terminal region. The predicted template modeling (pTM) and interface pTM (ipTM) scores are indicated in the figure. The low ipTM score is attributed to a high degree of structural disorder.

(**C**) EMSA showing the binding of GST-MYB40 (MYB40), GST-NSP1 (NSP1), and GST-NSP2 (NSP2) to the *ChOMT1* promoter region (p1). Different protein combinations are indicated below the gel image. Competition assays with the unlabeled wild-type (WT) or mutant (mut) probes are shown in the last four lanes.

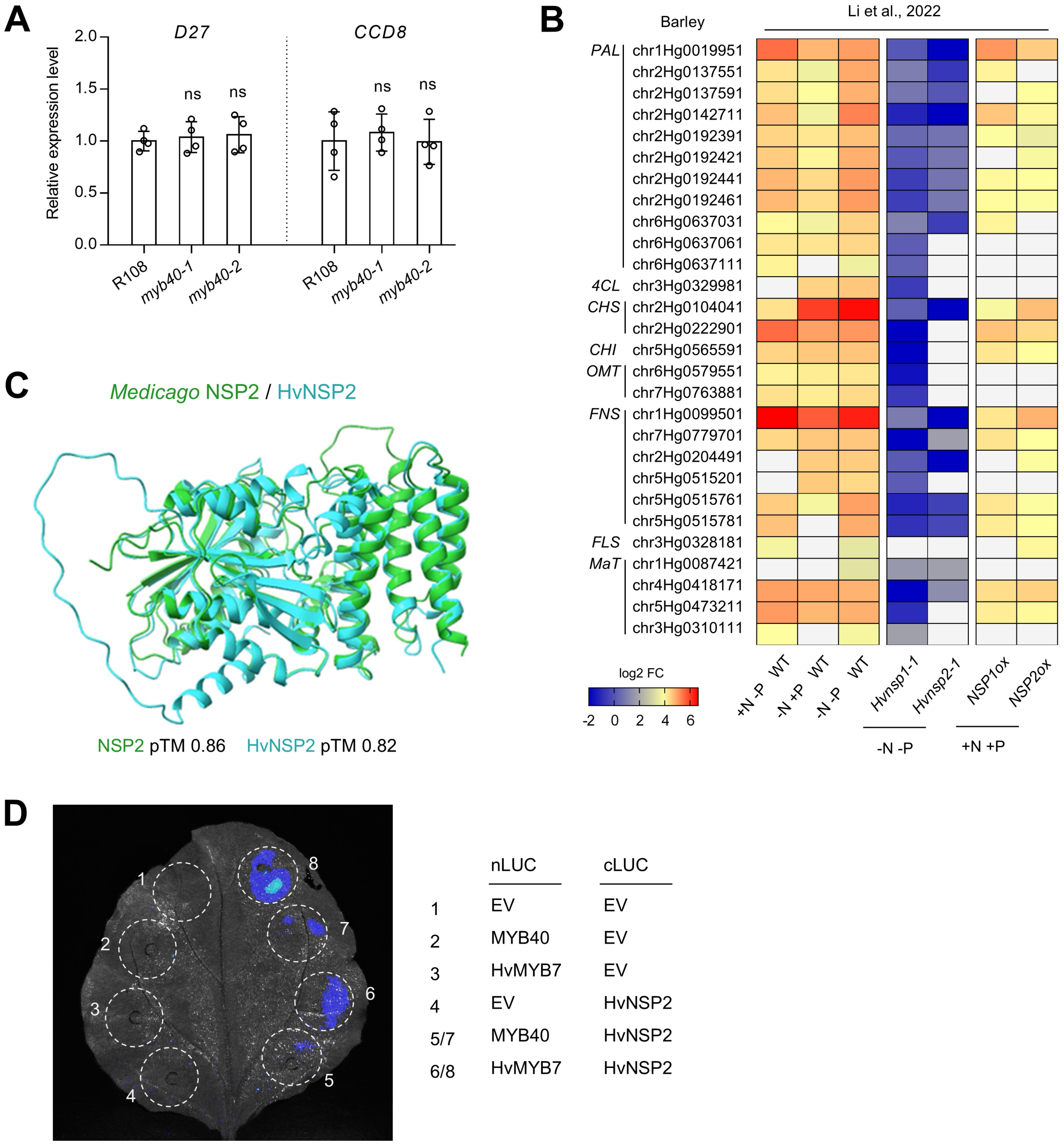

Figure S5. Flavonoid regulation by an NSP2-MYB module. Related to Figure 5.

(**A**) Expression levels of two strigolactone biosynthetic genes in R108, *myb40-1* and *myb40-2* roots. *D27*, *DWARF27*. *CCD8*, *Carotenoid Cleavage Dioxygenase 8*. Data are mean ± SD. Significant differences were determined by Student's *t*-test.

(**B**) Heatmaps showing selected phenylpropanoid and flavonoid biosynthetic genes regulated by *NSP1*/*NSP2* in response to nitrogen (N) and phosphorus (P) starvation, and activated by *NSP* overexpression in barley. Genes involved in phenylpropanoid and flavonoid biosynthetic pathways are annotated. +N-P, -N+P, and -N-P represent the expression of these genes in wild type plants by comparing −N or/and −P conditions to +N+P. The *nsp* mutants show gene expression in *nsp* mutants compared to wild-type plants under nutrient depletion, while *NSPox* shows *NSP* overexpression roots compared to wild type under nutrient-replete condition. The

concentrations used were defined as follows: -N-P, no NO^{3-} and no PO_4^{3-}; −N+P, no NO^{3-} and 0.5 mM PO_4^{3-}; +N-P, 5 mM NO^{3-} and no PO_4^{3-}; +N+P, 5 mM NO^{3-} and 0.5 mM PO_4^{3-}.

(**C**) Structural superposition of *Medicago* NSP2 (green) and barley HvNSP2 (cyan), predicted by AlphaFold3. The conserved structure domains of HvNSP2 (amino acid 108-508) and NSP2 (amino acid 122-566) showed high similarity. The predicted template modeling (pTM) scores are indicated in the figure.

(**D**) Split luciferase (LUC) complementation assays between HvNSP2 and HvMYB7. The N-terminal fragment of LUC (nLUC)-tagged HvMYB7 was co-infiltrated into *N. benthamiana* leaves along with the C-terminal fragment of LUC (cLUC)-tagged HvNSP2. HvMYB7 (a homolog of *Medicago* MYB40) and HvNSP2 showed interaction in *N. benthamiana*.

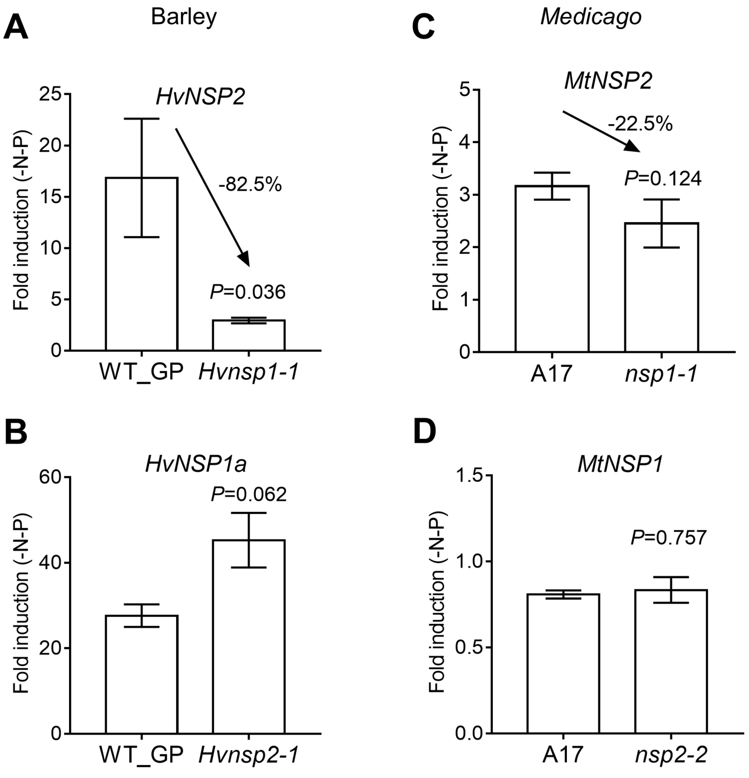

Figure S6. Comparative analysis of *NSP1* and *NSP2* expression patterns between *nsp1* and *nsp2* mutants under nutrient starvation. Related to Figure 1 and Figure 5.

(**A**) Expression levels of *HvNSP2* in wild-type (Golden Promise) and *Hvnsp1-1* mutant under nutrient-deficient (-N-P) conditions.

(**B**) Expression levels of *HvNSP1* in wild-type (Golden Promise) and *Hvnsp2-1* mutant under -N-P conditions.

(**C**) Expression levels of *MtNSP2* in wild-type (A17) and *nsp1-1* mutant under -N-P conditions.

(**D**) Expression levels of *MtNSP1* in wild-type (A17) and *nsp2-2* mutant under -N-P conditions.

The concentrations used were defined as follows: -N-P, no NO^{3-} and no PO_4^{3-}; −N+P, no NO^{3-} and 0.5 mM PO_4^{3-}; +N-P, 5 mM NO^{3-} and no PO_4^{3-}; +N+P, 5 mM NO^{3-} and 0.5 mM PO_4^{3-}. Data are mean ± SEM and the raw data from a previous study[S2]. Significant differences were determined by Student's *t*-test.

Primer name	Sequence (from 5'- to -3')	Description
GUS-MYB40-F	CGCCGAATTCCCGGGGATCCAAATAAGTCGCACAGACAAATCA	promoter-GUS
GUS-MYB40-R	AACTAGTCAGATCTACCATGGTTTTATTAGTTAGAAGAGAAGACAG	promoter-GUS
GUS-ChOMT1-F	CGCCGAATTCCCGGGGATCCTATACTGTATTAACAGGTATTTTTC	promoter-GUS
GUS-ChOMT1-R	AACTAGTCAGATCTACCATGGTTTTTGATTAGAACTAATGATTTTTTC	promoter-GUS
GUS-CYP75B1-F	CGCCGAATTCCCGGGGATCCTATGGAATGATGTGATTAGAGGTG	promoter-GUS
GUS-CYP75B1-R	AACTAGTCAGATCTACCATGGATTTGGTGAATTTTGACTTTGTTTTG	promoter-GUS
GUS-IMaT7-F	CGCCGAATTCCCGGGGATCCAATGAGCTCAATATTCTTACTAAC	promoter-GUS
GUS-IMaT7-R	AACTAGTCAGATCTACCATGGGGTTTATCTATTTCATCAAGAAG	promoter-GUS
EF1-qF	CTTTGCTTGGTGCTGTTTAGATGG	qPCR
EF1-qR	ATTCCAAAGGCGGCTGCATA	qPCR
CHS1-qF	GACAAGATTTAGTGGTAGTG	qPCR
CHS1-qR	GAACTCTTGCATTTTTGTTAT	qPCR
ChOMT1-qF	GGTTAGACCGAATGTTGCGTT	qPCR
ChOMT1-qR	CTTTCATCAGGGACAAGGTATTTT	qPCR
PAL1-qF	GGTCAATGTAAAGTAAAGTCCATG	qPCR
PAL1-qR	GATTAATGGTTGCCATGTTTGGAG	qPCR
IMaT7-qF	TGATGAAATAGATAAACCATGGCAC	qPCR
IMaT7-qR	TTGGTTTGGATGAATCACTAGGC	qPCR
CYP75B1-qF	ACATGTGTTGGAGGAGCACATTG	qPCR
CYP75B1-qR	TTGGTGTCACAGGATGAAGCCTC	qPCR
MYB41-qF	TAGATGTGGGAAAAGTTGTAGAC	qPCR
MYB41-qR	GAAGATGTGTGTTCCAGTAGTTC	qPCR
MtD27-qF	GAGATGATATTCGGCCAGGAAC	qPCR
MtD27-qR	GCATGGTTTTTCTTAGCCTTGC	qPCR
MtCCD8-qF	GAAGATGGGAGGGTAACTGCTG	qPCR
MtCCD8-qR	AGAACATCTTCGCCGTTAAATG	qPCR
ChOMT1-P1-qF	ACTACAAGAGACAATATTTGCAGG	ChIP-qPCR
ChOMT1-P1-qR	CATACTTTAAAAGTGGAGAATTCG	ChIP-qPCR
ChOMT1-P2-qF	CACTGTGTTTAACTTGAATTGT	ChIP-qPCR
ChOMT1-P2-qR	ATGGCACGTACAATCACTTGCC	ChIP-qPCR
ChOMT1-P3-qF	CTAACTGATAAGTGATCACTAC	ChIP-qPCR
ChOMT1-P3-qR	CCACATACTTTGTGTGCACGATC	ChIP-qPCR
ChOMT1-P4-qF	GTAGGTGAATCCTCCTAGCACA	ChIP-qPCR
ChOMT1-P4-qR	ATATTGTGCTCTATTTATAGGC	ChIP-qPCR
ChOMT1-P5-qF	CCTATAAATAGAGCACAATATTG	ChIP-qPCR
ChOMT1-P5-qR	CATTTTTTGATTAGAACTAATGAT	ChIP-qPCR
EMSA adaptor	AGCCAGTGGCGATAAG	EMSA
BK-MYB40-F	ATGGCCATGGAGGCCGAATTCatgGGAAGAACTCCTTGTTGTTC	Y2H
BK-MYB40-R	CGCTGCAGGTCGACGGATCCCGCTAATAAGGACTGAGCAAAAGA	Y2H
MYB40-NLUC-F	GGACGAGCTCGGTACCCGGGATCCatgGGAAGAACTCCTTGTTGTTC	Spilt-LUC
MYB40-NLUC-R	GGGACGCGTACGAGATCTGGTCGACCGCTAATAAGGACTGAGCAAAAG	Spilt-LUC
NSP2-CLUC-F	tcgtacgcgtcccggggcggtaccATGGATTTGATGGACATGGATGC	Spilt-LUC

NSP2-CLUC-R	CGAACGAAAGCTCTGCAGGTCGACCTATAAATCAGAATCTGAAGAAG	Spilt-LUC
HvMYB7-NLUC-F	ggacgagctcggtacccgggatccATGGGGAGGATGAGGAAGGAAGG	Spilt-LUC
HvMYB7-NLUC-R	GGGACGCGTACGAGATCTGGTCGACTAGCGGCATGTCCACAGAGTT	Spilt-LUC
HvNSP2-CLUC-F	tcgtacgcgtcccggggcggtaccATGGACGTGACCATGGAGGACG	Spilt-LUC
HvNSP2-CLUC-R	CGAACGAAAGCTCTGCAGGTCGACGACTAGCGCGTAAGCAGGTTCC	Spilt-LUC
pGreenII-0800-F	GTCGACGGTATCGATAAGCTT	Dual LUC
pGreenII-0800-R	GCTCTAGAACTAGTGGATCC	Dual LUC
Tnt1-R1	TGTAGCACCGAGATACGGTAATTAACAAGA	Genotyping
Tnt1-R2	AGTTGGCTACCAATCCAACAAGGA	Genotyping
NF21386-F	GTTACCGGCTGTCTGTCAATTC	Genotyping
NF21386-R	CAAATCCGAGTAGTGTTCGTGATTG	Genotyping
NF9316-F	GGTGGTTGATGCAGGGCTTCTTAG	Genotyping
NF9316-R	CTTCTGGTACTTTGGTTATGACTTTG	Genotyping
GST-NSP1-F	CCGCGTGGATCCCCGGAATTCATGACTATGGAACCAAATCCAAC	Fusion protein
GST-NSP1-R	GATGCGGCCGCTCGAGTCGACCTACTCTGGTTGTTTATCCAGTT	Fusion protein
GST-NSP2-F	CCGCGTGGATCCCCGGAATTCATGGATTTGATGGACATGGATGC	Fusion protein
GST-NSP2-R	GATGCGGCCGCTCGAGTCGACCTATAAATCAGAATCTGAAGAAG	Fusion protein
GST-MYB40-F	CCGCGTGGATCCCCGGAATTCATGGAAGAACTCCTTGTTGTTC	Fusion protein
GST-MYB40-R	GATGCGGCCGCTCGAGTCGACTCACGCTAATAAGGACTGAGCA	Fusion protein

Table S1. Primers used in this study. Related to STAR Methods.

Supplemental References

S1. Liu, Z., Yang, J., Long, Y., Zhang, C., Wang, D., Zhang, X., Dong, W., Zhao, L., Liu, C., Zhai, J., and Wang, E. (2023). Single-nucleus transcriptomes reveal spatiotemporal symbiotic perception and early response in *Medicago*. *Nat Plants* 9, 1734-1748. 10.1038/s41477-023-01524-8.

S2. Li, X.R., Sun, J., Albinsky, D., Zarrabian, D., Hull, R., Lee, T., Jarratt-Barnham, E., Chiu, C.H., Jacobsen, A., Soumpourou, E., et al. (2022). Nutrient regulation of lipochitooligosaccharide recognition in plants via *NSP1* and *NSP2*. *Nat Commun* 13, 6421. 10.1038/s41467-022-33908-3.
